# Genetics is a major determinant of expression of the human hepatic uptake transporter OATP1B1, but not of OATP1B3 and OATP2B1

**DOI:** 10.1186/gm405

**Published:** 2013-01-11

**Authors:** Anne T Nies, Mikko Niemi, Oliver Burk, Stefan Winter, Ulrich M Zanger, Bruno Stieger, Matthias Schwab, Elke Schaeffeler

**Affiliations:** 1 Dr. Margarete Fischer-Bosch Institute of Clinical Pharmacology, Auerbachstrasse 112, 70376 Stuttgart, Germany, and University of Tübingen; 2 Department of Clinical Pharmacology, University of Helsinki and HUSLAB Helsinki University Central Hospital, FI-00014 Helsinki, Finland; 3 Division of Clinical Pharmacology and Toxicology, University Hospital Zürich, Rämistrasse 100, 8091 Zürich, Switzerland; 4 Department of Clinical Pharmacology, Institute of Experimental and Clinical Pharmacology and Toxicology, University of Tübingen, Otfried-Müller-Strasse 45, 72076 Tübingen, Germany

## Abstract

**Background:**

Organic anion transporting polypeptide (OATP) 1B1, OATP1B3, and OATP2B1 (encoded by *SLCO1B1, SLCO1B3, SLCO2B1*) mediate the hepatic uptake of endogenous compounds like bile acids and of drugs, for example, the lipid-lowering atorvastatin, thereby influencing hepatobiliary elimination. Here we systematically elucidated the contribution of *SLCO *variants on expression of the three hepatic OATPs under consideration of additional important covariates.

**Methods:**

Expression was quantified by RT-PCR and immunoblotting in 143 Caucasian liver samples. A total of 109 rare and common variants in the *SLCO1B3-SLCO1B1 *genomic region and the *SLCO2B1 *gene were genotyped by MALDI-TOF mass spectrometry and genome-wide SNP microarray technology. *SLCO1B1 *haplotypes affecting hepatic OATP1B1 expression were associated with pharmacokinetic data of the OATP1B1 substrate atorvastatin (*n *= 82).

**Results:**

Expression of OATP1B1, OATP1B3, and OATP2B1 at the mRNA and protein levels showed marked interindividual variability. All three OATPs were expressed in a coordinated fashion. By a multivariate regression analysis adjusted for non-genetic and transcription covariates, increased OATP1B1 expression was associated with the coding *SLCO1B1 *variant c.388A > G (rs2306283) even after correction for multiple testing (*P *= 0.00034). This held true for haplotypes harboring c.388A > G but not the functional variant c.521T > C (rs4149056) associated with statin-related myopathy. c.388A > G also significantly affected atorvastatin pharmacokinetics. *SLCO *variants and non-genetic and regulatory covariates together accounted for 59% of variability of OATP1B1 expression.

**Conclusions:**

Our results show that expression of OATP1B1, but not of OATP1B3 and OATP2B1, is significantly affected by genetic variants. The *SLCO1B1 *variant c.388A > G is the major determinant with additional consequences on atorvastatin plasma levels.

## Background

The organic anion transporting polypeptides 1B1 (OATP1B1, encoded by the *SLCO1B1 *gene), OATP1B3 (*SLCO1B3*), and OATP2B1 (*SLCO2B1*) are major uptake transporters on the sinusoidal membrane of human hepatocytes. They mediate the influx of endogenous compounds such as bile salts, bilirubin glucuronides, thyroid hormones and steroid hormone metabolites, and clinically frequently used drugs like statins, HIV protease inhibitors, and the anti-cancer agents irinotecan or methotrexate [1-5]. The importance of OATP transporters for hepatobiliary uptake is emphasized by the Rotor syndrome, which is a two-gene disorder caused by the complete combined deficiency of OATP1B1 and OATP1B3 [6].

Numerous clinical studies support the relevance of common but also rare *SLCO1B1 *missense variants altering either the pharmacokinetics or drug response of OATP1B1 substrates [4,7,8]. The common variant c.521T > C (rs4149056; Val174Ala) is highlighted by a genome-wide association study (GWAS) suggesting an increased risk for simvastatin-induced myopathy in variant carriers [9]. Reduced hepatic uptake of the OATP1B1 substrates atorvastatin and rosuvastatin is supported by *in vitro *experiments using cell lines stably expressing the c.521T > C variant [2,10]. Moreover, the *in vivo *disposition of endogenous and/or xenobiotic substances, including drugs, is also affected by *SLCO1B3 *and *SLCO2B1 *variants [11].

Irrespective of well-established data on functional consequences of *SLCO *variants, the contribution of *SLCO *variants to the interindividual variability of hepatic expression of OATP transporters is still unknown. Although cholestasis has been recognized as an additional determinant of OATP1B1 and OATP1B3 expression [12], a comprehensive analysis including *SLCO *variants as well as non-genetic and regulatory covariates is presently lacking. Similar to recent work related to the hepatic uptake transporters OCT1 and OCT3 [13], we therefore investigated the impact of > 100 *SLCO *variants as well as non-genetic and regulatory covariates on the interindividual variability of hepatic OATP1B1, OATP1B3, and OATP2B1 expression. The transcription factors hepatocyte nuclear factor (HNF)1α, farnesoid X receptor (FXR), liver X receptor (LXR)α, specificity protein 1 (Sp1), aryl hydrocarbon receptor (AhR), constitutive androstane receptor (CAR), and HNF3β were selected because literature data indicate they are involved in regulation of the three hepatic *SLCO *genes with potential consequences on variability of expression [14-21]. The novel finding that the *SLCO1B1 *missense variant Asn130Asp (rs2306283) alters hepatic protein expression most effectively is supported by pharmacokinetic data of atorvastatin in a study of healthy volunteers. *SLCO *variants and non-genetic and regulatory covariates accounted for 59% of variability of hepatic OATP1B1 expression.

## Materials and methods

### Human liver samples

Liver tissue and corresponding blood samples were collected from patients undergoing liver surgery at the Department of General, Visceral, and Transplantation Surgery (University Medical Center Charité, Berlin, Germany) as described previously [13,22,23]. Tissue samples had been examined by a pathologist; only histologically normal liver tissue was used for further studies. For each patient, detailed information was available regarding age, sex, smoking status, alcohol consumption, presurgery medication, indication for liver resection, and presurgery liver serum parameters. Samples from patients with hepatitis, cirrhosis, or chronic alcohol use were excluded. A total of 143 liver samples from which high quality RNA and complete documentation could be obtained were finally included (Table S1 in Additional file 1). The study was approved by the ethics committees of the Charité, Humboldt University (Berlin, Germany) and the University of Tübingen (Tübingen, Germany) in accordance with the principles of the Declaration of Helsinki. Written informed consent was obtained from each patient.

### Atorvastatin pharmacokinetics *in vivo*

Atorvastatin pharmacokinetic variables were obtained from 82 healthy volunteers (Table S2 in Additional file 1) as previously described [24-26]. Briefly, volunteers ingested a single 20 mg dose of atorvastatin (Lipitor; Pfizer/Gödecke, Karlsruhe, Germany). No other drugs or grapefruit products were consumed prior to atorvastatin administration [25]. The study was approved by the Ethics Committee of the Hospital District of Helsinki and Uusimaa, Finland. Written informed consent was obtained from the participants.

### Selection of genetic variants and genotyping strategies

Genomic DNA was purified from EDTA blood samples with the QIAmp DNA Blood MiniKit (Qiagen, Hilden, Germany). All samples were genotyped for 109 genetic variants (Figure S1 and Table S3 in Additional file 1). We selected 58 genetic variants of the *SLCO1B3-SLCO1B1 *genomic region (chromosome 12) and the *SLCO2B1 *gene (chromosome 11) from the National Center for Biotechnology Information database (dbSNP build 129) based on functional criteria and/or frequency distribution. Genotyping was performed by matrix-assisted laser desorption/ionization time-of-flight mass spectrometry (MALDI-TOF MS) using the MassARRAY Compact system (Sequenom, San Diego, CA, USA) or by 5'-nuclease assays (ABI Prism 7900 Sequence Detection System, Applied Biosystems/Life Technologies, Carlsbad, CA, USA). Moreover, 51 additional variants of the *SLCO1B3-SLCO1B1 *genomic region and the *SLCO2B1 *gene were genotyped using the HumanHap300v1.1 chip data set (Illumina, San Diego, CA, USA) as described previously [27] (NCBI Gene Expression Omnibus series GSE39036, GSE32504). For MALDI-TOF MS and TaqMan analysis, about 10% of samples were re-genotyped as quality control resulting in 100% concordance. Missing calls in MALDI-TOF MS and TaqMan genotyping were re-typed, resulting in final call rates of 99%. Laboratory staff were blinded to the case status of liver samples. Details of primers and genotyping assays are available upon request.

### RNA isolation and quantification

High-quality total RNA was extracted from liver samples and reverse-transcribed as described [13]. mRNA was quantified by TaqMan technology (Additional file 1).

### Quantification of OATPs in human liver samples

OATP proteins were quantified in membrane fractions from the liver samples by immunoblot analyses using previously characterized antibodies [28,29] (Additional file 1).

### Generation of HEK cells stably expressing OATP2B1 and missense variants

Human embryonic kidney (HEK) cells were transfected with constructs encoding human *SLCO2B1 *or the missense variants c.601G > A (rs35199625; GenBank: NM_007256.4 as reference), c.935G > A (rs12422149), and c.1457C > T (rs2306168) (Additional file 1).

### Immunofluorescence microscopy

Cryosections of liver samples were immunostained for OATP1B1 and OATP2B1 using previously characterized antibodies [29,30]. Images were taken with a confocal laser scanning microscope (Additional file 1).

### Transport studies and prediction of functional effects

Transport of atorvastatin, rosuvastatin, and estrone sulfate by OATP2B1 and variants was measured using stably-transfected HEK cells (Additional file 1). Functional effects of OATP2B1 missense variants were calculated using four different algorithms (Additional file 1).

### Statistics

Hardy-Weinberg equilibrium calculations [31,32] were used to compare observed and expected allele and genotype frequencies. Linkage disequilibrium analysis of the *SLCO1B3-SLCO1B1 *genomic region and the *SLCO2B1 *gene was performed with Haploview [31] using results from our study population of 143 Caucasians. Haplotype analysis was performed with R-package haplo.stats-1.4.4 [33] (R-2.13.0).

R-Package SNPassoc-1.6-0 was applied to study associations between each variant and OATP expression, with correction for non-genetic (Table 1) and transcription factors (HNF1α, Sp1, AhR, LXRα, FXR, CAR, HNF3β). Multivariate linear models and step-wise model selection were used to determine the fraction of variance in OATP expression explained by non-genetic, genetic, and transcription covariates. All statistical tests were two-tailed and statistical significance was defined as *P *< 0.05. Where indicated, *P*-values were adjusted for multiple testing according to Holm [34] (see Additional file 1 for detailed information).

**Table 1:** Multivariate analysis of hepatic OATP expression in relation to 10 non-genetic factors in the total sample set of 143 human livers

	*P*-value					
	
Non-genetic factor	*SLCO1B1 *mRNA	OATP1B1 protein	*SLCO1B3 *mRNA	OATP1B3 protein	*SLCO2B1 *mRNA	OATP2B1 protein
Sex	0.903	0.766	0.661	0.463	0.623	0.072
Age	0.502	0.630	0.466	0.060	0.711	0.947
Smoking habit	0.534	0.454	0.173	0.731	0.214	0.278
Alcohol consumption	0.037	0.124	0.076	0.904	0.001	0.206
Pre-surgery drugs	0.294	0.786	0.638	0.401	0.567	0.168
Diagnosis	0.458	0.046	0.238	0.101	0.506	0.066
Bilirubin	0.846	0.108	0.348	0.194	0.997	0.847
γ-Glutamyl transferase	0.363	0.820	0.548	0.448	0.461	0.547
Cholestasis	0.286	0.020	0.008	0.021	0.467	0.232
C-reactive protein	0.039	0.070	0.873	0.132	0.520	0.466

## Results

### Hepatic OATP expression

**Figure 1:**
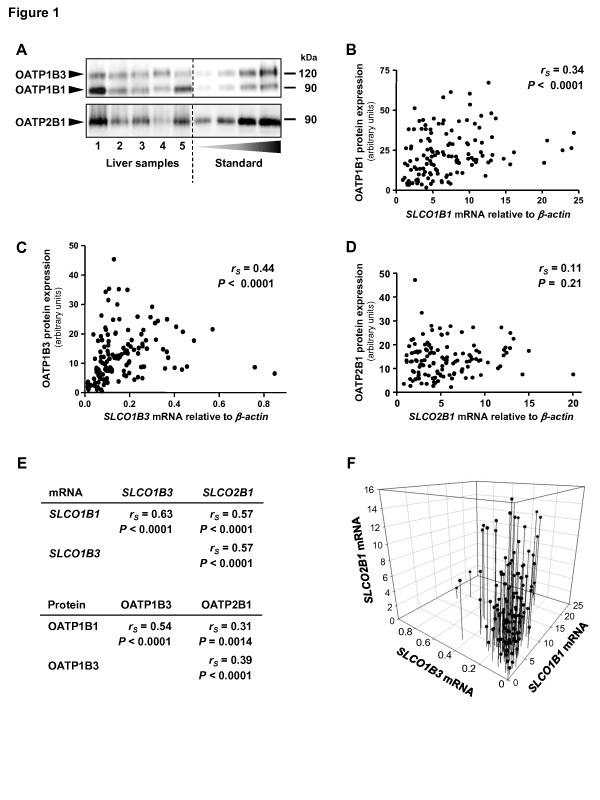
**Hepatic expression of OATP1B1, OATP1B3 and OATP2B1 varies between individuals, but is coordinately regulated within one individual**. **(a) **Representative immunoblot of liver membrane fractions (20 μg) quantified relative to a standard liver sample. **(b-d) **Correlation analyses of OATP protein and *SLCO *mRNA levels in human liver samples. **(e, f) **Correlation analyses of all three OATPs showed a coordinated expression of *SLCO1B1, SLCO1B3*, and *SLCO2B1 *mRNA and OATP1B1, OATP1B3, and OATP2B1 protein levels in the non-cholestatic liver sample set. r_S_, Spearman rank-order correlation coefficient.

OATP1B1, OATP1B3, and OATP2B1 mRNA and protein expression varied considerably within the 143 liver samples and were not normally distributed (Figure 1; Figure S2 in Additional file 1). mRNA and protein expression weakly correlated for *SLCO1B1*/OATP1B1 and *SLCO1B3*/OATP1B3, but not for *SLCO2B1*/OATP2B1 (Figure 1b-d). Marked interindividual variability was also obtained when only non-cholestatic liver samples (*n *= 117) were analyzed (Table S4A in Additional file 1). Moreover, of interest, *SLCO1B1, SLCO1B3*, and *SLCO2B1 *mRNA levels were significantly correlated with each other, which held true to a lesser extent for protein levels (Figure 1e, f).

### Impact of non-genetic factors on OATP expression

Multivariate linear regression analyses showed no association between OATP expression and sex, age, smoking habit, presurgery medication, bilirubin levels, or γ-glutamyl transferase levels (Table 1). Alcohol consumption was significantly associated with reduced *SLCO1B1 *and *SLCO2B1 *mRNA levels. Pathophysiologically increased C-reactive protein levels were associated with reduced *SLCO1B1 *mRNA levels. As expected from previous studies [12], OATP1B1 and OATP1B3 protein levels were significantly lower in cholestatic liver samples. When analyzing only the non-cholestatic samples, alcohol consumption was again significantly associated with reduced *SLCO2B1 *mRNA levels (Table S5 in Additional file 1).

### *SLCO *genetic variants and frequencies

**Figure 2:**
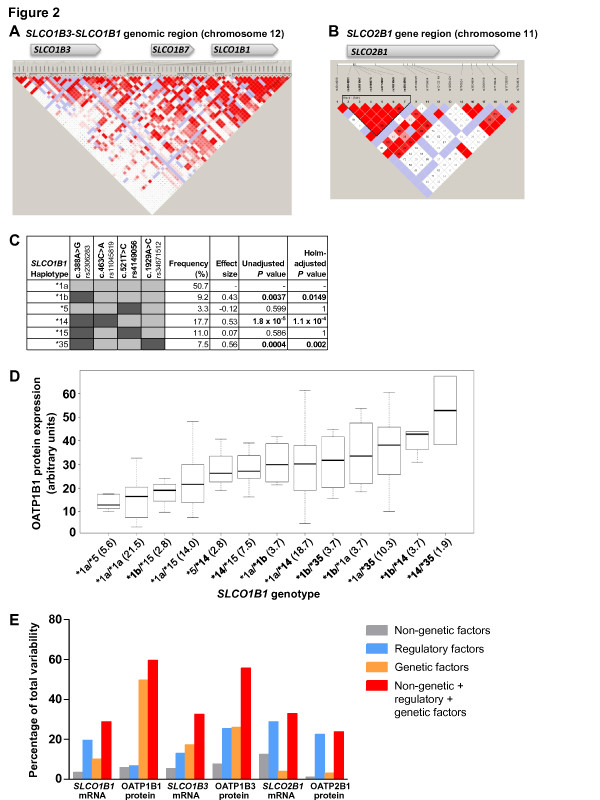
***SLCO *genetic variants affect expression of hepatic OATPs**. **(a, b) **Pairwise linkage disequilibrium map of the *SLCO1B3-SLCO1B1 *genomic region (a) and the *SLCO2B1 *gene (b) including all variants detected in the 143 livers. Coloring corresponds to the standard Haploview (D'/LOD) [31]. **(c,d) ***SLCO1B1 *haplotypes, calculated based on the four missense variants present in the liver cohort (c.388A > G, c.463C > A, c.521T > C, c.1929A > C) and also previously described as key variants [8,80], affect OATP1B1 protein expression in the non-cholestatic liver samples (*n *= 117). Only haplotypes with frequencies ≥ 2% are given. The effect sizes indicate differences of OATP1B1 expression compared to reference haplotype SLCO1B1*1a. Boldface: significant *P*-values. (d) *SLCO1B1 *genotypes (percentage genotype frequencies in brackets) are sorted by median OATP1B1 protein expression. *SLCO1B1 *alleles harboring only variant c.388A > G and not c.521T > C (*1b, *14, *35, boldface) confer considerably higher OATP1B1 expression in a gene-dose dependent manner. Horizontal line indicates median; boxes indicate the 25th to 75th percentiles; whiskers indicate non-outlier range. **(e) **Percentage of interindividual variability of expression of OATPs in the non-cholestatic liver samples apportioned to non-genetic factors (gray), regulatory factors (blue), and *SLCO *variants (orange), and to the combination of all three categories (red) was calculated using multivariate linear regression analyses and stepwise model selection.

Table S3 in Additional file 1 specifies all 109 common and rare variants genotyped, including additional information on location and allele frequencies. We detected 83 variants in the 143 liver samples, 66 variants in the *SLCO1B3-SLCO1B1 *genomic region and 17 variants in the *SLCO2B1 *gene (Figure S1 in Additional file 1). No deviations from Hardy-Weinberg equilibrium were observed. Notably, nearly complete linkage disequilibrium was observed for several variants in *SLCO1B1 *(Figure 2a).

### *SLCO*/OATP genotype-phenotype correlation analyses

In order to exclude confounding by cholestasis, all subsequent analyses were performed with the non-cholestatic liver samples (*n *= 117). Multivariate linear regression models were used to analyze associations between variants with frequencies ≥ 1% in the *SLCO1B3-SLCO1B1 *genomic region and the *SLCO2B1 *gene (Figure S1 in Additional file 1) and the expression of OATP1B1, OATP1B3, and OATP2B1. Models were corrected for eight non-genetic covariates (Table S6 in Additional file 1) and the regulatory factors HNF1, LXR, FXR, HNF3, Sp1, CAR, and AhR previously suggested by literature data as transcriptional regulators of at least one *SLCO *gene [14-21]. Interindividual variability of the expression of these transcription factors is given in Table S4B in Additional file 1.

Considering the additive genetic model, 38 variants in the *SLCO1B3-SLCO1B1 *genomic region correlated with OATP1B1 or OATP1B3 expression, and 3 variants in the *SLCO2B1 *gene with OATP2B1 expression (Table S6 in Additional file 1). After multiple testing correction, 16 variants in the *SLCO1B3-SLCO1B1 *genomic region were still significantly associated with OATP1B1 expression. Notably, for the OATP1B1 protein, variant c.388A > G (Asn130Asp, rs2306283) showed the lowest *P*-value (*P *= 0.00034) whereas the variant c.521T > C (Val174Ala, rs4149056), associated with statin-induced myopathy [9,35,36], did not show any association. Stratification of baseline characteristics by carriers of c.388A > G (dominant model) did not show any significance.

Additionally, we calculated haplotypes for the non-cholestatic liver samples using different strategies. Firstly, haplotypes were calculated for genetic variants of *SLCO1B1, SLCO1B3*, and *SLCO2B1 *detected in the liver samples (Table S3 in Additional file 1). Only variants with a frequency ≥ 1% were included. Significant associations with substantial effect sizes between haplotypes and expression were only found for OATP1B1 protein and *SLCO2B1 *mRNA expression, even after correction for multiple testing (Tables S7 to S9 in Additional file 1). Secondly, previously reported *SLCO1B1 *haplotypes containing only the variants c.388A > G, c.463C > A, c.521T > C and c.1929A > C were calculated (Figure 2c). The haplotypes *1b (*P *= 0.0037), *14 (*P *= 1.8 × 10^-5^), and *35 (*P *= 0.0004), including the c.388A > G but not the c.521T > C variant, were significantly associated with increased OATP1B1 protein expression, even after multiple testing correction. These haplotypes exhibited substantial effect sizes (*1b, 0.43; *14, 0.53; *35, 0.56) and clustered with samples of highest median OATP1B1 expression (Figure 2d). OATP1B1 protein levels also increased with the number of *1b, *14, and *35 alleles (Figure S3A in Additional file 1).

Thirdly, we calculated haplotypes covering all variants of the *SLCO1B3-SLCO1B1 *genomic region (Table S10 in Additional file 1). Two haplotypes, which included the *SLCO1B1 *variant c.388A > G, were significantly associated with increased OATP1B1 protein levels and showed substantial effect sizes for the correlation (H-cluster04, 0.62; H-cluster12, 0.59).

### Contribution of genetic, non-genetic, and transcription factors to variable OATP expression

Interindividual variability of hepatic OATP1B1 protein expression was substantially explained by *SLCO1B1 *variants (50%) while non-genetic (8%) and transcription factors (7%) contributed only to a minor extent (Figure 2e). Notably, 59% of variability could be apportioned to the combination of all three categories (that is, genetic, non-genetic, transcription factors). Total variance of OATP1B3 protein expression was explained to 55% by these categories but only 24% of variance of OATP2B1 protein expression could be apportioned to the three categories.

### Atorvastatin pharmacokinetics

**Figure 3:**
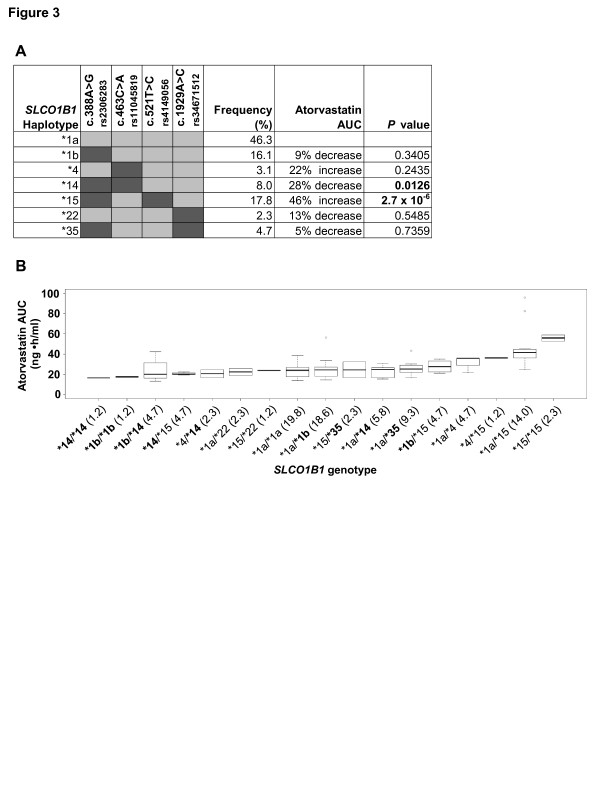
***SLCO1B1 *haplotypes affect atorvastatin pharmacokinetics in 82 healthy volunteers**. **(a) ***SLCO1B1 *haplotypes calculated on the presence of the four previously described key variants and change in atorvastatin area under the plasma concentration-time curve (AUC), corrected for body weight, in comparison with the reference haplotype SLCO1B1*1a. Haplotypes are given with a frequency > 2%. **(b) ***SLCO1B1 *genotypes (percentage genotype frequencies in brackets) are sorted by median atorvastatin AUC. *SLCO1B1 *alleles harboring only variant c.388A > G and not c.521T > C (boldface) confer considerably lower AUCs with lowest levels in homozygous variant or compound heterozygous carriers for the SLCO1B1*14 or *1b alleles. Horizontal line indicates the median; boxes indicate 25th to 75th percentiles; whiskers indicate non-outlier range.

*SLCO1B1 *haplotypes for all 82 volunteers were calculated considering the *SLCO1B1 *variants c.388A > G, c.463C > A, c.521T > C and c.1929A > C. Seven different haplotypes with a frequency distribution ≥ 2% were detected (Figure 3a; Table S11 in Additional file 1). A linear regression analysis including body weight as covariate indicated that the haplotypes *1b and *14, both harboring c.388A > G but not c.521T > C, were associated with decreased atorvastatin area under the plasma concentration-time curve (AUC) compared to the reference haplotype (*1a), reaching statistical significance for *14 (*P *= 0.0126). Notably, homozygous carriers of *1b and *14 showed the lowest atorvastatin AUC (Figure 3b). In contrast, haplotype *15, harboring c.388A > G and c.521T > C, was associated with an increased atorvastatin AUC (*P *= 2.7 × 10^-6^; Figure 3a) and an increased atorvastatin C_max _(*P *= 0.031; Table S11 in Additional file 1). Here, homozygous carriers for *15 showed highest AUC for the total study cohort (Figure 3b). Atorvastatin AUC also decreased with the number of *1b and *14 alleles (Figure S3B in Additional file 1).

In order to identify whether the effects of variant c.388A > G may be confounded by variant c.521T > C, we conducted multivariate models including both variants simultaneously. The variant c.388A > G was significantly associated with decreased AUC for atorvastatin (*P *= 0.00066, recessive model) whereas the variant c.521T > C resulted in an increased AUC (*P *= 2.6 × 10^-10^, additive model). Of note, these data held true when the interaction effect of c.388A > G and c.521T > C was included into the model (c.388A > G, *P *= 0.00535; c.521T > C, *P *= 3.0 × 10^-7^; interaction effect, *P *= 0.67).

### Statin transport by OATP2B1 and its missense variants

The *SLCO2B1 *missense variants c.601G > A, c.935G > A and c.1457C > T were the only ones present in our liver cohort (Table S3 in Additional file 1). Although they had no effect on expression (Figure 2e), functional consequences of these variants could not be excluded with certainty. We therefore stably expressed the three variants separately in HEK cells. The variant transfectants showed correct immunolocalization of OATP2B1 in the plasma membrane (Figure S4A in Additional file 1). Only c.1457C > T but not c.601G > A or c.935G > A showed reduced uptake of atorvastatin, rosuvastatin and the prototypical substrate estrone sulfate (Figure S5 in Additional file 1). The calculated maximal uptake rate of atorvastatin was significantly lower for c.1457C > T and tended to be lower for rosuvastatin (Table S12 in Additional file 1). In contrast, the K_m _value of c.935G > A was significantly higher for atorvastatin (Table S12 in Additional file 1). Sinusoidal hepatocyte membrane immunostaining of OATP2B1 in liver cryosections from homozygous variant carriers for c.935AA was similar to OATP2B1-reference (Figure S4B in Additional file 1). Moreover, four independent computational tools did not correctly predict experimental uptake data (Table S13 in Additional file 1). Altogether, the uptake studies indicate some functional consequences, which needs validation by *in vivo *studies.

## Discussion

This first systematic analysis of the human membrane transporters OATP1B1, OATP1B3, and OATP2B1 on the interindividual variability of hepatic expression revealed a major contribution of *SLCO1B1 *variants on OATP1B1 protein expression. Expression of OATP1B3 or OATP2B1 was apparently not influenced by genetic variants. Most strikingly, the *SLCO1B1 *missense variant c.388A > G resulted in the strongest association with increased OATP1B1 protein expression whereas the c.521T > C variant, linked to statin-related myopathy [9,35,36], did not alter protein levels. This was observed for both the non-cholestatic livers (*n *= 117; Figure 2c, d) as well as the total liver set of 143 samples (Table S14 in Additional file 1), indicating that c.388A > G alters OATP1B1 expression independent of cholestasis. The observation that variant c.388A > G is associated with highest OATP1B1 protein expression is corroborated by our haplotype analysis since homozygous or compound heterozygous carriers of haplotypes *1b, *14, or *35, harboring c.388A > G but not c.521T > C, showed also the highest OATP1B1 protein levels. A mislocalization of OATP1B1 protein in genotyped livers for variant c.388A > G could be excluded by immunolocalization analyses (Figure S6 in Additional file 1).

The results of the liver study are confirmed by functional data of the pharmacokinetic healthy volunteer study since the *14 haplotype was associated with a significantly decreased atorvastatin plasma AUC in contrast to the *15 haplotype, harboring both c.388A > G and c.521T > C, and resulting in an increased AUC. Thus, the *SLCO1B1 *variant c.388A > G is an important determinant of OATP1B1 expression and function, independent of the c.521T > C variant. In line with *in vitro *reports [37,38], one may suggest that the increased clearance by OATP1B1 c.388A > G is mainly caused by a higher protein expression. The increased hepatic atorvastatin uptake in c.388A > G carriers is also supported by haplotype data from previous studies indicating that only c.388A > G was associated with significant lower AUCs for the OATP1B1 substrates pravastatin and repaglinide [39-41]. Moreover, variant c.388A > G carriers showed a significantly improved response to statins measured by low-density lipoprotein cholesterol levels [42,43] and were even protected from statin-induced side effects [42]. Of note, however, the effect of the c.388A > G variant seems to be substrate-specific since the AUC was not altered, for example, for rosuvastatin [44], underscoring the requirement to perform pharmacokinetic studies for each OATP1B1 drug separately. Mechanistically, we suggest that variant c.388A > G modifies mRNA secondary structure, resulting in an alteration of translation efficiency as previously shown for the CFTR membrane protein [45]. Indeed, computational theoretical modeling provided evidence that the *SLCO1B1 *mRNA secondary structures are altered only by haplotypes *1b, *14, and *35 harboring the c.388A > G variant (Figure S7 in Additional file 1).

In line with the localization of the OATP1B1*15 protein in the cell membrane using stably expressing HEK cells [38], a mislocalization of the OATP1B1 protein in livers genotyped for *15 haplotype was not found. The increased atorvastatin AUC in carriers of the *15 haplotype in our pharmacokinetic study revealed impaired atorvastatin uptake, indicating that the variant OATP1B1*15 protein is associated with decreased intrinsic transport activity. This observation is supported by previous studies using oocytes that revealed significantly reduced atorvastatin uptake by OATP1B1*15 in comparison with the reference sequence [46].

Another novel observation of our study is the coordinated expression of OATP1B1, OATP1B3, and OATP2B1 in human liver. The coordinate expression may be explained by regulatory networks considering previously identified transcription factors like HNF1α, Sp1, AhR, LXRα, FXR, CAR, and HNF3β [14-21]. Since HNF1α confers basal promoter activity of *SLCO1B1 *and *SLCO1B3 *and functional binding sites have been detected in the promoter regions of both genes [14,17], we analyzed the impact of *HNF1α *on *SLCO *mRNA expression. Because exon_1e transcripts of *SLCO2B1 *are highly abundant in human liver [47] and the exon_1e promoter region has so far not been analyzed, we identified three high-scoring putative HNF1α binding sites in the exon_1e promoter (Methods and Figure S8A in Additional file 1) by *in silico *analysis. Electromobility shift assays confirmed *in vitro *binding of HNF1α to each of the three identified motifs, thereby indicating that the exon_1e transcript of *SLCO2B1 *is regulated by HNF1α in liver. Binding of HNF1α to any of the three sites in the exon_1e promoter of *SLCO2B1 *appeared to be weaker than binding to the respective motifs in the *SLCO1B1 *and *SLCO1B3 *gene promoters (Figure S8B in Additional file 1).

Among several clinical and demographic factors, including age, sex, alcohol consumption, smoking habit, and presurgery medication, only cholestasis resulted in significantly reduced OATP1B1 and OATP1B3 protein levels considering the total set of 143 liver samples (Table 1). This is in line with previous data since cholestasis in cases of primary biliary cirrhosis or progressive familial intrahepatic cholestasis is associated with reduced OATP1B1 and OATP1B3 expression [12] and the concerted down-regulation of both bile salt uptake transporters is considered as a protective mechanism against hepatocellular injury caused by cytotoxic bile acids. Cholestasis-related down-regulation of OATP1B1 and OATP1B3 might be of clinical relevance regarding drug response to several well-established OATP substrates, for example, lopinavir [48] and irinotecan [49].

Moreover, one aim of our study was to elucidate systematically the contribution of genetic, non-genetic and regulatory factors for the prediction of the interindividual variability of the expression of hepatic OATP transporters. Generally, the interindividual variability of expression of hepatic OATPs is considerably higher than variation of the hepatic intrinsic clearance of drugs that are substrates for OATP transport proteins as reported by clinical studies [50]. This discrepancy is, however, not surprising since the hepatic clearance is the sum of a number of processes and factors - for example, involvement of different drug transporters for one substrate drug, short-term regulation of transporters (for example, by protein kinases), differences in retrieval of transporters from the plasma membrane, and multiple binding sites on transporters. Our multivariate analyses only using non-cholestatic liver samples revealed that the combination of genetic, non-genetic, and transcription factors explained 59% and 55% of variability of OATP1B1 and OATP1B3 protein expression, respectively, while OATP2B1 protein variance remained largely unaffected (24%). Several reasons might explain the poor prediction of interindividual variability of hepatic OATP2B1 expression, although systematic ascertainment of covariates in our liver bank was guaranteed [51] (Table S1 in Additional file 1). Other non-genetic factors like cytokines [52] may affect OATP2B1 expression. Moreover, unidentified transcription factors, genetic variation in those genes as well as epigenetics such as DNA methylation [53] or regulation by microRNA [54] may contribute to the interindividual variability of OATP2B1.

## Conclusions

In our comprehensive work on the expression of hepatic OATPs we show for the first time a major contribution of *SLCO1B1 *genetics to the interindividual variability of OATP1B1 protein expression. The *SLCO1B1 *variant c.388A > G strongly affects OATP1B1 expression with additional functional consequences on atorvastatin plasma levels. Further work is warranted to identify underlying mechanisms for the so far unexplained interindividual variability of hepatic OATP1B3 and OATP2B1 expression.

## Abbreviations

AhR: aryl hydrocarbon receptor; AUC: area under the plasma concentration-time curve; CAR: constitutive androstane receptor; FXR: farnesoid X receptor; HEK: human embryonic kidney cells; HNF: hepatocyte nuclear factor; LXR: liver X receptor; MALDI-TOF MS: matrix-assisted laser desorption/ionization time-of-flight mass spectrometry; OATP: organic anion transporting polypeptide; SLC: solute carrier; Sp1: specificity protein 1.

## Competing interests

The authors declare that they have no competing interests.

## Authors' contributions

MS was responsible for study design and drafting the manuscript. ATN and ES were responsible for study design and drafting the manuscript, and collected, analyzed and interpreted data. MN collected, analyzed and interpreted data. OB analyzed and interpreted data. Statistical analysis was performed by SW. UMZ and BS provided samples and reagents and critically revised the manuscript for important intellectual content. All authors have read and approved the manuscript for publication.

## Supplementary Material

Additional data file 1 **Additional methods, tables and figures **[55-79]. Click here for file
