# Supplementary material for: Genetics is a major determinant of expression of the human hepatic uptake transporter OATP1B1, but not of OATP1B3 and OATP2B1
Source: Genome Med. 2013 Jan 11;5(1):1. doi: 10.1186/gm405 (PMC3706890; doi:10.1186/gm405)

Additional file 1

Nies *et al.*: Genetics is a major determinant of expression of the human hepatic uptake transporter OATP1B1, but not of OATP1B3 and OATP2B1

Methods: **page 4 – 12**

Tables S1 to S15: **page 13 – 35**

Figures S1 to S8: **page 36 – 43**

Information on additional data file 1

File name: Nies et al_additional file 1.pdf

File format: pdf

Title of data: Additional methods, tables and figures

Description of data: The following additional data are available with the online version of this paper. Methods: additional description of methods. RNA isolation and quantification in human liver samples was performed using previously described methods [13,22,51,55]. HEK cells stably expressing OATP2B1 and missense variants were generated using previously described methods [56]. Antibodies and methods used for immunoblot and immunofluorescence analyses [13,22,28,29,57,58]. Transport studies were performed as described previously [59,60]. HNF1 binding sites in the *SLCO2B1* promoter [47] were predicted *in silico* and electromobility shift assays were performed as described [61-64]. Functional effects of OATP2B1 missense variants were predicted with the algorithms PolyPhen-2 [65,66], SIFT [67,68], PMut [69,70], and SNPs3D [71,72]. Secondary mRNA structures were predicted with MFold [73-75]. Expression data for transcription factors were extracted from our previous work [27]. Table S1: population demographics and other annotations of the human liver samples and published criteria for liver serum parameters [76,77]. Table S2: population demographics and atorvastatin pharmacokinetic variables of the healthy volunteer study. Table S3: genotyping methods, minor allele frequencies and other annotations of the genotyped *SLCO* variants [78]. Table S4: variability of OATP and transcription factor expression in the non-cholestatic liver samples. Table S5: multivariate analysis of hepatic OATP expression in the non-cholestatic liver samples in relation to non-genetic factors. Table S6: multivariate analysis of hepatic OATP expression in relation to genetic variants, corrected for non-genetic covariates and regulatory factors considering different genetic models. Table S7: *SLCO1B1* haplotype frequencies and their effects on OATP1B1 expression. Table S8: *SLCO1B3* haplotype frequencies and their effects on OATP1B3 expression. Table S9: *SLCO2B1* haplotype frequencies and their effects on OATP2B1 expression. Table S10: haplotype frequencies of the *SLCO1B3-SLCO1B1*

genomic region and effects on expression. Table S11: *SLCO1B1* haplotypes and atorvastatin pharmacokinetics. Table S12: kinetic parameters for atorvastatin, rosuvastatin, and estrone sulfate uptake by OATP2B1. Table S13: *in silico* prediction of functional effects of OATP2B1 missense variants. Table S14: *SLCO* genetic variants affect expression of hepatic OATPs. Table S15: sequences of oligonucleotide primers and TaqMan probes. Figure S1: selection of SLCO genetic variants for genotyping, genotyping methods, variants detected and used for statistical analysis [27,79]. Figure S2: systematic analysis of OATP expression in human liver samples. Figure S3: *SLCO* genetic variants affect expression of hepatic OATPs and atorvastatin pharmacokinetics. Figure S4: immunolocalization of OATP2B1 and missense variants in transfected HEK cells and in human liver. Figure S5: functional characterization of OATP2B1 and 3 missense variants using atorvastatin, rosuvastatin, and the prototypical substrate estrone sulfate. Figure S6: immunolocalization of OATP1B1 in cryosections from genotyped human liver samples. Figure S7: prediction of secondary *SLCO1B1* mRNA structures. Figure S8: transcriptional regulation of *SLCO* expression.

Methods

RNA isolation and quantification in human liver samples

As previously described [13,22,51,55], all samples were derived from patients who received hepatectomy using an identical sample processing (e.g. timing of liver excision and preservation, storage of tissue samples, sample preparation) to minimize confounding by pre-analytical issues.

RNA quality of each sample was controlled with the Agilent 2100 Bioanalyzer using the RNA6000 Nano Lab Chip Kit (Agilent, Santa Clara, CA) as described [13]. Each sample showed strong 18S and 28S bands and no RNA degradation.

We designed primer-probe sets for quantification of *SLCO1B1*, *SLCO1B3*, and *SLCO2B1* (Table S15). Each set was specific for its respective target gene as tested with plasmids containing *SLCO1B1*, *SLCO1B3*, or *SLCO2B1* cDNA. Detection of genomic DNA was prevented by designing probes that contain sequences of two exons. Probes were 5'-labeled with the fluorescent reporter dye 6-carboxyfluorescein and 3'-labeled with a minor groove binding nonfluorescent quencher. *β-actin* was analyzed with a predeveloped assay (Applied Biosystems, Foster City, CA, USA). Standard curves for the *SLCO1B1*, *SLCO1B3*, *SLCO2B1*, and *β-actin* assay were calculated by using serial dilutions of known amounts of plasmid cDNA. Each target mRNA level was normalized to *β-actin* mRNA levels.

Cloning of human *SLCO2B1* cDNA, construction of *SLCO2B1* variant vectors, and generation of stably transfected HEK cells

The full-length cDNA encoding human OATP2B1 was amplified from human liver and cloned into the expression vector pcDNA3.1/V5-His-TOPO (Invitrogen, Carlsbad, CA, USA). The following primers were used for amplification: hSLCO2B1-for 5'-GCAGTCATGGGACCCAGG-3' and hSLCO2B1-rev 5'-TCACACTCGGGAATCCTCTG-3'. The coding sequence of the cloned *SLCO2B1* cDNA was identical to the reference sequence NM_007256.4 except for having nucleotide T at mRNA position 1991 (NCBI SNP ID rs61555831, Ser532Ser). The vector with the reference sequence was used as template for

mutagenesis. Variant *SLCO2B1* vectors encoding OATP2B1-c.601G>A (rs35199625, NP_009187.1:p.Val201Met), OATP2B1-c.935G>A (rs12422149, NP_009187.1: p.Arg312Gln), or OATP2B1-c.1457C>T (rs2306168, NP_009187.1:p.Ser486Phe) were constructed using the QuikChange multi site-directed mutagenesis kit (Stratagene/Agilent Technologies, Santa Clara, CA, USA). Vectors were sequenced to confirm presence of the intended variant and absence of any other nucleotide exchange in the *SLCO2B1* cDNA.

Human embryonic kidney 293 (HEK) cells (CRL-1573; American Type Culture Collection, Manassas, VA) were grown in DMEM (Sigma) supplemented with 10% fetal bovine serum (Sigma), 100 U/ml penicillin, and 100 µg/ml streptomycin (Lonza, Basel, Switzerland) at 37°C and 5% CO_2. HEK cells were transfected with Metafectene Pro (Biontex, München, Germany). Cell clones stably expressing OATP2B1 or variants were selected with 800 µg/ml G418 and screened for expression by immunoblot analysis and immunofluorescence microscopy as described [56]. HEK cells stably transfected with the empty pcDNA3.1(+) vector served as controls (HEK-Co). Cells were incubated with 5 mM butyrate 24 h before use to increase protein levels of recombinant transporters [56].

Antibodies

The rabbit polyclonal antibody against the carboxyl terminus of human OATP2B1 has been described previously [29]. The mouse monoclonal antibody for simultaneous detection of OATP1B1 and OATP1B3 by immunoblotting [28] was from Progen (clone mMDQ, Heidelberg, Germany). The mouse monoclonal antibody against dipeptidylpeptidase IV (DPPIV, CD26) was from Ancell Corp. (Bayport, MN, USA) and used to identify canalicular membranes as described previously [58]. Alexa Fluor546-conjugated goat anti-mouse IgG and Alexa Fluor488-conjugated goat anti-rabbit IgG were from Invitrogen (Carlsbad, CA).

Immunoblot analysis of OATP1B1, OATP1B3, and OATP2B1

Crude membrane fractions could be prepared from 132 human liver tissue samples. Membrane fractions from liver samples and transfected cells were prepared as described

[13] and stored at -80 °C. Crude membrane fractions were denatured for 30 min at 37 °C in Laemmli sample buffer and separated on 10% SDS/polyacrylamide gels. Proteins were transferred onto nitrocellulose membranes (Protran BA85, Whatman GmbH, Dassel, Germany) using a tank blotting system (Mini-Protean Tetracell, Biorad, München, Germany). Membranes were blocked for 1 h at room temperature with 5% (wt/vol) skim milk in TBS-T (140 mM NaCl, 20 mM Tris-HCl, pH 7.6, 0.1 % (wt/vol) Tween-20). Membranes were incubated for 16 h at 4 °C with the OATP2B1 antibody [29] (1:2,000 dilution), or with the monoclonal OATP1B1/OATP1B3 antibody [28] (1:50 dilution). After washing with TBS-T, membranes were incubated with the corresponding horseradish peroxidase-conjugated antibodies (1:5,000 dilution; Santa Cruz Biotechnology) for 60 min at room temperature and finally washed with TBS-T. Membranes were developed with enhanced chemoluminescence detection solution (Supersignal WestDura, Pierce, Rockford, IL, USA) and chemoluminescence was measured with a charge-coupled device camera (Fuji LAS-1000, Raytest, Straubenhardt, Germany).

Quantification of OATP proteins in human liver samples

Membrane fractions from human liver (20 µg protein) were separated on SDS/PAGE gels and blotted as described above. Nitrocellulose membranes were cut at the 50 kDa marker band. The upper membrane half was incubated for 16 h at 4 °C with the monoclonal OATP1B1/OATP1B3 antibody [28] or the OATP2B1 antibody [29] and the lower half with the anti-β-actin antibody (1:5,000 dilution; clone AC-15, Sigma, St. Louis, MO, USA), respectively. Subsequent steps were as described above. Protein expression levels were analyzed semi-quantitatively by densitometry with AIDA 2.31 software (Raytest). Calibration curves were obtained by co-analyzing on each gel serial dilutions of known amounts of total protein from a defined liver sample (3.75, 7.5, 15, and 30 µg protein in right part of Figure 1A). As shown before [28], with the monoclonal OATP1B1/OATP1B3 antibody OATP1B3 can be detected at a band of ~120 kDa and OATP1B1 at ~90 kDa, both of which were therefore used for densitometric analysis. A weak band at ~75 kDa was also visible, probably

corresponding to less-glycosylated OATP1B1. As previously shown, because only the 90kDa-band of OATP1B1 is considered to correspond to glycosylated cell surface-expressed OATP1B1 [57] and is thereby involved in drug uptake, we only quantified this band. OATP2B1 was detected at ~85 kDa as described before [29]. Calibration curves were generated by densitometric analysis of each band. Interblotting variability was tested and considered acceptable as previously described for the reproducibility of expression analyses of other transporters [13,22].

Immunofluorescence microscopy of tissue samples and cells

Cryosections of human liver tissue (5 µm) were prepared and fixed with methanol as described [13]. HEK cells transfected with OATP2B1-reference or missense variant c.601G>A, c.935G>A, or c.1457C>T were grown for 2 days on glass slides and also fixed with methanol at –20 °C for 10 min. Cryosections or cells were incubated with the primary antibody and subsequently the corresponding secondary antibodies for 1 h. Antibodies were diluted in PBS as follows: polyclonal rabbit anti-OATP2B1 [29] 1:100, polyclonal rabbit anti-OATP1B1 [30] 1:100, monoclonal mouse anti-CD26 1:400, monoclonal mouse anti-CD68 1:100, fluorochrome-conjugated secondary antibodies 1:300. Images were taken with a confocal laser scanning microscope (TCS NT Confocal System, Leica Microsystems, Wetzlar, Germany).

Transport studies

HEK cells (500000 cells/well) were seeded into 24-well cell culture plates (Greiner Bio-One, Frickenhausen, Germany) and grown for 48 h. Cell adherence was improved by coating plates with poly-L-lysine (Biochrom AG, Berlin, Germany) prior to cell plating. All uptake studies were carried out at 37 °C as described [60]. For uptake measurements, cells were firstly washed with uptake buffer (142 mM NaCl, 12.5 mM hydroxyethylpiperazine ethanesulfonic acid, 5 mM KCl, 1 mM KH_2PO_4, 1.5 mM $CaCl_2$, 1.2 mM $MgSO_4$, 5 mM glucose, pH 7.3) [59] prewarmed to 37 °C. Uptake was initiated by replacing this solution with

uptake buffer containing different concentrations of atorvastatin, rosuvastatin, or estrone sulfate and tracer amounts of ^{3}H-labeled atorvastatin (15 nM), rosuvastatin (25 nM), or estrone sulfate (20 nM). [5-^{3}H]atorvastatin (specific activity 20 Ci/mmol) and [^{3}H(G)]rosuvastatin (10 Ci/mmol) were from American Radiolabeled Inc. (St. Louis, MO), [6,7-^{3}H(N)]estrone sulfate (54.3 Ci/mmol) was from PerkinElmer (Boston, MA).

Uptake was stopped within the linear uptake phase for each substrate, i.e. after 2 min for atorvastatin, 1 min for rosuvastatin, and 30 sec for estrone sulfate, by removing the uptake buffer and immediately washing the cells 3 times with ice-cold uptake buffer. Cells were lysed with 0.2% SDS and intracellular radioactivity was determined by liquid scintillation counting (Hidex 300SL TDCR liquid scintillation counter, Turku, Finland). Protein content of lysed cells was determined in 25-µl aliquots using the bicinchoninic acid assay as described [58].

To compare the transport activity of different OATP2B1 variants, expression of each variant was normalized to that of OATP2B1-reference sequence and the corrected protein levels were used for calculating uptake values. Protein levels were determined by densitometric analysis of immunoblots using the rabbit polyclonal antibody against the carboxyl terminus of human OATP2B1 [29].

For each substrate and concentration, 3 individual experiments each with 3 parallel measurements were performed. Uptake values were corrected for uptake into vector-transfected control cells. For each individual experiment, K_m and V_{max} values for statin or estrone sulfate uptake were determined by fitting the Michaelis-Menten equation to uptake measurements using Prism 5 software (GraphPad Software, La Jolla, California, USA). Data points shown in Figure S5 and kinetic parameters (Table S12) are the means of the 3 individual experiments ± SE.

Identification of HNF1 binding sites and electromobility shift assays

2.6 kb of the sequence upstream of exon_1e of human *SLCO2B1* [47] were retrieved from Ensembl release 64. Putative HNF1 binding sites were identified by MATCH-1.0 (Biobase,

Wolfenbüttel, Germany) using the predefined liver-specific profile and further scored using the HNF1-weighted half-site matrix [61]. The 3 highest-scoring sites were selected for *in vitro* binding analysis by electromobility shift assays. Annealing of respective oligonucleotides, radioactive labeling, and analysis of binding reactions were performed as described previously [62]. For this, mouse HNF1α protein was synthesized *in vitro* using the expression plasmid pcDNA3-mHNF1α [63] and the TNT T7 Quick Coupled Transcription/Translation System (Promega, Madison, WI, USA).

Oligonucleotides containing known functional HNF1 binding sites were as follows: *SLCO1B1* sense, 5'-GATCCAAGGTGGTTAATCATCACTGGACTTA-3'; *SLCO1B1* antisense, 5'-GATCTAAGTCCAGTGATGATTAACCACCTTG-3'; *SLCO1B3* sense, 5'-GAT CCAAGATGGTTAATCATCATTGGACTCA-3'; *SLCO1B3* antisense, 5'-GATCTGAGTC CAATGATGATTAACCATCTTG-3'. Oligonucleotides containing putative HNF1 sites of the promoter region of exon_1e of *SLCO2B1* were as follows: site A sense, 5'-GAT CCATGTGCTTGAATATTTATCAGGAAA-3'; site A antisense, 5'-GATCTT TCCTGATAAATATTCAAGCACATG-3'; site B sense, 5'-GATCCACCTGG CTAATTTTTTGCATTTGTA-3'; site B antisense, 5'-GATCTACAAATGCAAA AAATTAGCCAGGTG-3'; site C sense, 5'-GATCCAGGCTGGTTTATTATTTTTTAATCA-3'; site C antisense, 5'-GATCTGATTAAAAAATAATAAACCAGCCTG-3'. Annealing of respective sense and antisense single-stranded oligonucleotides to double-stranded oligonucleotides and radioactive labeling was performed as described previously [62]. Binding reactions and gel electrophoresis were performed as described elsewhere [64]. Retarded complexes were quantified with the BAS-1800 II phosphor-storage scanner (Fuji, Kanagawa, Japan) and AIDA software (Raytest, Straubenhardt, Germany).

Prediction of functional effects using 4 algorithms

Prediction of functional effects of OATP2B1 missense variants was calculated with PolyPhen-2 [65,66], SIFT (Sorting Intolerant from Tolerant) Human Protein DB [67,68], PMut [69,70], and SNPs3D [71,72]. The default settings were used for executing all programs.

Computational structural analyses

Secondary mRNA structure was predicted with MFold [73-75] using the default settings and the complete *SLCO1B1* mRNA sequence for analysis. Only the respective optimal structure is shown.

Statistical methods

Data distribution was tested by the method of Kolmogorov and Smirnov. Because data sets were not normally distributed, significance of correlations was tested by correlation tests on the basis of the Spearman rank-order correlation coefficient (r_s). Prism 5 software (GraphPad Software Inc., La Jolla, CA) was used for these calculations.

All other analyses were performed with statistics software R-2.13.0 [33]. To meet the Gaussian assumption of the different statistical methods described in more detail below, we first transformed mRNA/protein expression data by taking third roots. Data of the atorvastatin pharmacokinetic variables “area under the curve (AUC)”, “peak plasma concentration (C_{max})”, and “half-life (t½)” were log-transformed. Data distributions were checked using normal quantile–quantile plots. Genetic variants with minor allele frequencies (MAFs) <1% were excluded from further analyses.

Multivariate linear regression models were used to estimate the effects of the 10 non-genetic factors (sex, age, smoking habit, alcohol consumption, pre-surgery drugs, diagnosis, bilirubin, γ-glutamyl transferase, cholestasis, C-reactive protein) on OATP mRNA and protein expression in all 143 liver samples (results in Table 1). Additionally, a similar analysis was performed in the subset of non-cholestatic liver samples (n=117) to estimate the effect of the 8 non-genetic factors sex, age, smoking habit, alcohol consumption, pre-surgery drugs, diagnosis, bilirubin, and γ-glutamyl transferase on OATP mRNA and protein expression (results in Table S5).

R-package SNPassoc-1.6-0 was applied to study associations between each genetic variant and OATP mRNA/protein expression, with correction for the 8 non-genetic factors (sex, age, smoking habit, alcohol consumption, pre-surgery drugs, diagnosis, bilirubin, γ-

glutamyl transferase) and the mRNA levels of 7 transcription factors (HNF1α, Sp1, AhR, CAR, FXR, LXRα, HNF3β transcript variant 1 NM_021784, two different probes for HNF3β transcript variant 2 NM_153675) (results in Table S6) in the 117 liver samples. Expression levels of the selected transcription factors were included since literature data indicate transcriptional regulation of at least one *SLCO* gene by those factors [14-19,21]. Expression data were extracted from previous work [27]. Four different genetic models were considered: codominant, dominant, recessive, and additive. In the codominant, dominant, and recessive models, genotypic data are treated as categorical variables. The codominant model compares homozygote carriers of the major allele vs. heterozygotes vs. homozygote carriers of the minor allele. The dominant model tests homozygote carriers of the major allele vs. heterozygotes + homozygote carriers of the minor allele. The recessive model tests homozygote carriers of the major allele + heterozygotes vs. homozygote carriers of the minor allele. For the additive model, a numeric variable is created with homozygote carriers of the major allele = 0, heterozygotes = 1, and homozygote carriers of the minor allele = 2, assuming a linear allele-dose effect. To increase statistical power, genetic variants having less than 3 homozygote carriers of the minor allele were investigated in the dominant model only.

Multivariate linear models and stepwise model selection were used to determine the fraction of variance in mRNA or protein expression explained by non-genetic factors and genetic variants in the 117 non-cholestatic liver samples (results in Fig. 2E). For this purpose, first multivariate linear models were defined with (a) all 8 non-genetic factors given in Table S5, (b) mRNA levels of the 7 transcription factors listed above, and (c) all genetic variants with $p<0.15$ in the analyses with correction for all non-genetic factors and transcription (regulatory) factors. Furthermore, the factors selected in (a), (b), and (c) were used to set up models with a combination of non-genetic covariates, genetic variants, and transcription factors. Stepwise model-selection using Akaike`s information criterion then resulted in final multivariate linear models containing non-genetic covariates, genetic variants, and transcription factors which explain mRNA or protein expression best. The

fraction of explained variance was then defined by the adjusted coefficient of determination (adjusted R^2) in order to account for the differing numbers of covariables in the models.

R-package haplo.stats-1.4.4 was used for haplotype analyses. The reference haplotype was defined as the most frequent haplotype. To analyze differences regarding OATP mRNA/protein expression between haplotypes having a frequency ≥2% and the reference haplotype, corrected for the 8 non-genetic factors (sex, age, smoking habit, alcohol consumption, pre-surgery drugs, diagnosis, bilirubin, γ-glutamyl transferase) and the 7 transcription factors (HNF1α, Sp1, AhR, CAR, FXR, LXRα, HNF3β), the general linear model capabilities of function haplo.glm were used and haplotypes were coded in the additive model. Given effect sizes are defined as differences to reference haplotype calculated on third root-transformed expression data (results in Figure 2C, D, Tables S7-S10). The same approach was applied to analyze associations between haplotypes having a frequency ≥2% and atorvastatin pharmacokinetic variables AUC, C_{max}, and half-life including weight as covariate (results in Figure 3, Table S11). In box-whisker plots, frequencies may not add up to 100% due to rounding.

Moreover, multivariate linear models incorporating both genetic variants c.388A>G and c.521T>C simultaneously (with and without interaction term) were used to determine whether one or both variants are significantly associated with atorvastatin AUC.

In addition, Fisher's exact tests and Wilcoxon-Mann-Whitney tests were used as appropriate to analyze whether there were differences between c.388AA, c.388AG or c.388GG genotypes and the 8 non-genetic factors in the liver samples or the 4 baseline characteristics of the atorvastatin study (age, sex, weight and height).

All statistical tests were 2-tailed and statistical significance was defined as P <0.05. Where indicated, P values were adjusted for multiple testing according to Holm's procedure [34] on the level of individual outcomes, i.e. mRNA expression separate from protein expression.

Table S1. Population demographics, serum parameters, and other annotations of the individuals from whom liver samples were obtained.

Subgroup		All samples (n=143)	Non-cholestatic samples (n=117)
**Sex**	Male	68	55
	Female	75	62
**Age**	≤70	117	99
	>70	26	18
**Smoking habit***	Non-smoker	110	89
	Smoker	29	26
**Alcohol consumption***	None	92	75
	≥1 times/week	46	39
**Pre-surgery drugs**	No	38	34
	Yes	105	83
**Diagnosis**	Primary liver cancer	43	75
	Metastases	81	27
	Other	19	15
**Total bilirubin***	≤1.2 mg/dL	121	112
	>1.2 mg/dL	20	5
**γ-GT***	≤36 U/L (female), ≤64 U/L (male)	82	78
	>36 U/L (female), >64 U/L (male)	55	36
**CrP***	≤8.2 mg/L	135	116
	>8.2 mg/L	6	1
**Cholestasis***	Non-cholestatic	117	117
	Cholestatic	23	0

*Numbers do not add up to 143 (all samples) or 117 (non-cholestatic samples) because of missing values
CrP, C-reactive protein; γ-GT, γ-glutamyl transferase; Upper limit of normal values were according to published criteria [76] and as follows: Total bilirubin: 1.2 mg/dL; γ-GT: 36 U/L (female), 64 U/L (male); CrP: 8.2 mg/L
Cholestasis was defined as previously described [13] according to published criteria [77].

Table S2. Population demographics and atorvastatin pharmacokinetic variables in 82 healthy Caucasian subjects.

	**Number**
**Sex**	
Male	45
Female	37
	**Median (range)**
**Age (years)**	22 (18-34)
**Height (cm)**	176 (154-192)
**Weight (kg)**	67.8 (48.0-92.0)
**Atorvastatin pharmacokinetic variables**	
C_{max} (ng/ml)	5.4 (0.64)
t_{max} (h)	0.5 (0.5-5.0)
t½ (h)	9.8 (0.31)
$AUC_{0-\infty}$ (ng•h/ml)	27.1 (0.44)

Pharmacokinetic data are geometric mean (geometric CV); t_{max} data are median (range).

C_{max}, peak plasma concentration; t_{max}, time to C_{max}; $t_{½}$, elimination half-life; $AUC_{0-\infty}$, area under the plasma concentration-time curve from 0 h to infinity

Table S3. Genotyping methods, minor allele frequencies (MAF), and other annotations of the 109 genotyped *SLCO* variants.

***SLCO1B3-SLCO1B1* genomic region**											
**SNP_ID**	**refSNP_ ID**	**dbSNP allele**	**Contig position (NT_009714.17)**	**Gene**	**Region**	**Protein residue**	**Contig allele**	**Minor allele**	**MAF**	**HWpval**	**Genotyping method**
1	rs1588918	G/T	13688927		intergenic region		G	G	0.338	0.9208	ILLUMINA
2	rs4762785	G/T	13705397		intergenic region		T	T	0.296	1.0	ILLUMINA
3	rs10841644	A/C	13706886		intergenic region		A	A	0.276	0.4886	ILLUMINA
4	rs7978273	C/T	13712289		intergenic region		T	C	0.121	1.0	ILLUMINA
5	rs1356148	C/T	13722403	SLCO1B3	5' near gene		T	C	0.304	0.2975	MALDI-TOF MS
6	rs11045521	A/G	13722879	SLCO1B3	5' near gene		G	A	0.164	0.8953	MALDI-TOF MS
7	rs1515766	C/T	13723259	SLCO1B3	5' near gene		T	C	0.101	0.3107	MALDI-TOF MS
8	rs10841661	C/T	13744956	SLCO1B3	intron		C	T	0.408	0.501	ILLUMINA
9	rs7310629	C/T	13745411	SLCO1B3	intron		C	T	0.174	0.7148	ILLUMINA
10	rs57325543	A/G	13768155	SLCO1B3	exon	Ile52Val (A>G)	A	G	0.0	1.0	MALDI-TOF MS
11	rs2900474	A/G	13770172	SLCO1B3	intron		C	C	0.137	0.5038	ILLUMINA
12	rs4149117	G/T	13771604	SLCO1B3	exon	Ser112Ala (T>G)	T	T	0.13	0.8572	MALDI-TOF MS
13	rs3764009	A/G	13774072	SLCO1B3	intron		C	C	0.136	0.4949	MALDI-TOF MS
14	rs77922474	A/G	13774167	SLCO1B3	exon	Asn151Ser (A>G)	A	G	0.0	1.0	MALDI-TOF MS
15	rs7306033	A/G	13774302	SLCO1B3	intron		A	G	0.16	1.0	ILLUMINA
16	rs7311358	A/G	13775884	SLCO1B3	exon	Met233Ile (G>A)	G	G	0.136	0.4949	MALDI-TOF MS
17	rs60140950	G/C	13788332	SLCO1B3	exon	Gly256Ala (G>C)	G	C	0.154	1.0	MALDI-TOF MS
18	rs61673910	A/G	13792667	SLCO1B3	exon	Gly437Ser (G>A)	G	A	0.0	1.0	MALDI-TOF MS
19	rs61736817	C/T	13793947	SLCO1B3	exon	Leu456Phe (C>T)	C	T	0.003	1.0	MALDI-TOF MS
20	rs72559743	G/T	13796542	SLCO1B3	exon	Gly522Cys (G>T)	G	T	0.0	1.0	MALDI-TOF MS
21	rs12299012	C/T	13796657	SLCO1B3	exon	Val560Ala (T>C)	T	C	0.0	1.0	MALDI-TOF MS
22	rs6487172	C/T	13804913	SLCO1B3	intron		T	T	0.323	0.4545	ILLUMINA
23	rs11045585	A/G	13805818	SLCO1B3	intron		A	G	0.136	0.1843	MALDI-TOF MS
24	rs3764006	C/T	13814493	SLCO1B3	exon	Gly611	G	G	0.113	0.4921	ILLUMINA
25	rs10841707	A/T	13830261	SLCO1B3	3' near gene		T	T	0.066	0.9452	MALDI-TOF MS
26	rs919842	A/G	13842400		intergenic region		G	G	0.063	0.874	ILLUMINA
27	rs10841712	A/G	13846507		intergenic region		A	A	0.372	0.4872	ILLUMINA
28	rs10841714	C/T	13858426		intergenic region		C	C	0.086	1.0	ILLUMINA
29	rs11519061	C/T	13880339		intergenic region		C	T	0.171	1.0	ILLUMINA
30	rs2417873	C/T	13926447		intergenic region		G	A	0.268	1.0	ILLUMINA
31	rs10505870	A/G	13932020	SLCO1B7	intron		G	A	0.338	0.326	ILLUMINA
32	rs1910186	A/C	13934041	SLCO1B7	intron		G	G	0.156	1.0	ILLUMINA
33	rs11045659	C/T	13942416	SLCO1B7	intron		C	T	0.218	0.5723	ILLUMINA
34	rs11045689	A/G	13961787	SLCO1B7	exon	Ala338Thr (G>A)	G	G	0.352	0.6814	ILLUMINA
35	rs1910169	C/T	13966885	SLCO1B7	intron		G	A	0.188	0.1692	ILLUMINA
36	rs11045773	A/G	14032426		intergenic region		A	G	0.209	0.1755	ILLUMINA
37	rs852550	A/G	14041891		intergenic region		C	C	0.067	0.9502	ILLUMINA
38	rs852549	A/C	14042013		intergenic region		T	T	0.063	0.874	ILLUMINA
39	rs4149013	A/G	14042534	SLCO1B1	5' near gene		A	G	0.046	1.0	TaqMan
40	rs17328763	C/T	14042694	SLCO1B1	5' near gene		T	C	0.189	0.1523	MALDI-TOF MS
41	rs4149015	A/G	14043446	SLCO1B1	5' near gene		G	A	0.042	1.0	MALDI-TOF MS
42	rs3829306	A/G	14052404	SLCO1B1	intron		C	T	0.046	1.0	ILLUMINA
43	rs11557087	A/G	14054660	SLCO1B1	exon	Thr10Ala (A>G)	A	G	0.0	1.0	MALDI-TOF MS
44	rs4149026	A/C	14075539	SLCO1B1	intron		A	C	0.385	0.8278	ILLUMINA
45	rs4149030	A/G	14076337	SLCO1B1	intron		A	G	0.479	0.4346	ILLUMINA
46	rs61760183	A/G	14085793	SLCO1B1	exon	Arg57Gln (G>A)	G	A	0.0	1.0	MALDI-TOF MS
47	rs56101265	C/T	14085840	SLCO1B1	exon	Phe73Leu (T>C)	T	C	0.0	1.0	MALDI-TOF MS
48	rs2291073	G/T	14085938	SLCO1B1	intron		T	G	0.089	1.0	ILLUMINA
49	rs56061388	C/T	14087653	SLCO1B1	exon	Val82Ala (T>C)	T	C	0.0	1.0	MALDI-TOF MS
50	rs4149038	A/G	14088366	SLCO1B1	intron		A	G	0.225	0.4731	ILLUMINA
51	rs17329885	C/T	14088689	SLCO1B1	intron		T	C	0.175	0.0885	ILLUMINA
52	rs964615	C/T	14089548	SLCO1B1	intron		T	T	0.105	1.0	ILLUMINA
53	rs2306283	C/T	14089862	SLCO1B1	exon	Asn130Asp (A>G)	A	G	0.427	0.8925	ILLUMINA
54	rs11045818	A/G	14089885	SLCO1B1	exon	Ser137	G	A	0.168	0.1176	MALDI-TOF MS
55	rs11045819	A/C	14089937	SLCO1B1	exon	Pro155Thr (C>A)	C	A	0.158	0.1893	MALDI-TOF MS
56	rs72559745	A/G	14089941	SLCO1B1	exon	Glu156Gly (A>G)	A	G	0.0	1.0	MALDI-TOF MS
57	rs4149056	C/T	14091673	SLCO1B1	exon	Val174Ala (T>C)	T	C	0.178	1.0	MALDI-TOF MS
58	rs4149057	C/T	14091723	SLCO1B1	exon	Leu191	T	T	0.405	0.6397	MALDI-TOF MS
59	rs2291075	C/T	14091749	SLCO1B1	exon	Phe199	C	T	0.43	0.5331	TaqMan
60	rs4603354	A/G	14091760	SLCO1B1	exon	Gly203Glu (G>A)	G	A	0.0	1.0	MALDI-TOF MS
61	rs2100996	C/T	14098321	SLCO1B1	intron		A	G	0.461	0.8225	ILLUMINA
62	rs11045834	C/T	14101220	SLCO1B1	intron		C	T	0.304	1.0	ILLUMINA
63	rs11045852	A/G	14110009	SLCO1B1	exon	Ile245Val (A>G)	A	G	0.0	1.0	MALDI-TOF MS
64	rs11045853	A/G	14110034	SLCO1B1	exon	Arg253Gln (G>A)	G	A	0.0	1.0	MALDI-TOF MS
65	no refSNP_ID	no dbSNF	14110151	SLCO1B1	exon	Ala292Val (C>T)	C	T	0.0	1.0	MALDI-TOF MS

***SLCO1B3-SLCO1B1* genomic region, continued**

SNP_ID	refSNP_ ID	dbSNP allele	Contig position (NT_009714.17)	Gene	Region	Protein residue	Contig allele	Minor allele	MAF	HWpval	Genotyping method
66	rs4149064	A/G	14110985	SLCO1B1	intron		A	G	0.057	1.0	ILLUMINA
67	rs59113707	C/G	14115613	SLCO1B1	exon	Phe400Leu (C>G)	C	G	0.0	1.0	MALDI-TOF MS
68	rs77468276	C/G	14115659	SLCO1B1	exon	Val416Leu (G>C)	G	C	0.0	1.0	MALDI-TOF MS
69	rs61176925	A/C	14115685	SLCO1B1	exon	Leu424Phe (A>C)	A	C	0.0	1.0	MALDI-TOF MS
70	rs56387224	A/G	14115707	SLCO1B1	exon	Asn432Asp (A>G)	A	G	0.0	1.0	MALDI-TOF MS
71	rs72559748	A/G	14118979	SLCO1B1	exon	Asp462Gly (A>G)	A	G	0.0	1.0	MALDI-TOF MS
72	rs59502379	G/C	14119057	SLCO1B1	exon	Gly488Ala (C>G)	G	C	0.0	1.0	TaqMan
73	rs4363657	C/T	14128846	SLCO1B1	intron		T	C	0.204	0.4976	ILLUMINA
74	rs72661137	T/G	14130307	SLCO1B1	exon	Leu543Trp (T>G)	T	G	0.0	1.0	MALDI-TOF MS
75	rs987839	C/T	14134962	SLCO1B1	intron		A	G	0.493	0.582	ILLUMINA
76	rs7966613	A/G	14139756	SLCO1B1	intron		A	G	0.379	0.9064	ILLUMINA
77	rs10841763	C/T	14142466	SLCO1B1	intron		T	C	0.387	0.9084	ILLUMINA
78	rs10841767	A/G	14146185	SLCO1B1	intron		A	G	0.195	0.6944	ILLUMINA
79	rs34671512	A/C	14152100	SLCO1B1	exon	Leu643Phe (A>C)	A	C	0.056	1.0	MALDI-TOF MS
80	rs56199088	A/G	14152135	SLCO1B1	exon	Asp655Gly (A>G)	A	G	0.0	1.0	MALDI-TOF MS
81	rs55737008	A/G	14152171	SLCO1B1	exon	Glu667Gly (A>G)	A	G	0.0	1.0	MALDI-TOF MS
82	rs4149087	G/T	14152686	SLCO1B1	3'UTR		T	G	0.444	0.8845	MALDI-TOF MS
83	rs11045891	A/C	14152696	SLCO1B1	3'UTR		A	C	0.188	0.8513	TaqMan
84	rs11045892	A/G	14152918	SLCO1B1	3' near gene		A	G	0.189	0.8019	MALDI-TOF MS
85	rs11045893	C/T	14152943	SLCO1B1	3' near gene		T	C	0.189	0.8019	MALDI-TOF MS
86	rs12372157	G/T	14153142	SLCO1B1	3' near gene		T	G	0.444	0.8845	MALDI-TOF MS
87	rs9804732	G/T	14164445		intergenic region		G	T	0.44	0.9681	ILLUMINA
88	rs7975087	A/C	14164956		intergenic region		A	C	0.264	0.3422	ILLUMINA
89	rs7311964	G/T	14173756		intergenic region		T	G	0.254	1.0	ILLUMINA

SLCO2B1

SNP_ ID	refSNP_ID	dbSNP allele	Contig position (NT_167190.1)	Gene	Region	Protein residue	Contig allele	Minor allele	MAF	HWpval	Genotyping method
1	rs4944989	A/G	20161091		intergenic region		G	A	0.025	1.0	ILLUMINA
2	rs4944991	C/T	20166581	SLCO2B1	5`near gene		T	C	0.234	0.9219	MALDI-TOF MS
3	rs2851067	T/G	20167123	SLCO2B1	5`near gene		T	T	0.434	0.1457	MALDI-TOF MS
4	rs4100076	A/C	20167211	SLCO2B1	5`near gene		A	C	0.21	0.7389	MALDI-TOF MS
5	rs2712807	A/G	20167676	SLCO2B1	5`near gene		G	G	0.199	0.2642	MALDI-TOF MS
6	rs2851069	C/T	20168151	SLCO2B1	5`UTR		T	T	0.434	0.1457	MALDI-TOF MS
7	rs4944994	C/T	20175683	SLCO2B1	intron		T	C	0.23	1.0	ILLUMINA
8	rs56837383	C/T	20179521	SLCO2B1	exon	Pro15Ser (C>T)	C	T	0.0	1.0	MALDI-TOF MS
9	rs35199625	A/G	20186165	SLCO2B1	exon	Val201Met (G>A)	G	A	0.007	1.0	MALDI-TOF MS
10	rs72559740	A/T	20186208	SLCO2B1	exon	Asp215Val (A>T)	A	T	0.0	1.0	MALDI-TOF MS
11	rs1789694	A/G	20187477	SLCO2B1	intron		C	C	0.264	0.1457	ILLUMINA
12	rs12422149	A/G	20189372	SLCO2B1	exon	Arg312Gln (G>A)	G	A	0.094	1.0	MALDI-TOF MS
13	rs10501421	A/G	20190183	SLCO2B1	intron		G	A	0.131	1.0	ILLUMINA
14	rs765824	A/C	20208290	SLCO2B1	intron		C	A	0.021	1.0	ILLUMINA
15	rs1621378	C/T	20210157	SLCO2B1	exon	Thr392Ile (C>T)	T	C	0.0	1.0	MALDI-TOF MS
16	rs3824904	C/T	20211530	SLCO2B1	intron		C	T	0.025	1.0	ILLUMINA
17	rs2306168	C/T	20213377	SLCO2B1	exon	Ser486Phe (C>T)	C	T	0.024	1.0	MALDI-TOF MS
18	rs7116044	A/C	20217749	SLCO2B1	intron		C	A	0.039	1.0	ILLUMINA
19	rs17133815	A/G	20219551	SLCO2B1	intron		G	A	0.035	1.0	ILLUMINA
20	rs7924924	C/T	20229007		intergenic region		T	C	0.043	1.0	ILLUMINA

Minor allele frequencies (MAF), Hardy-Weinberg *P* value (HWpval) and other annotations of 89 genotyped variants in the *SLCO1B3-SLCO1B1* genomic region (chromosome 12) and of 20 variants in the *SLCO2B1* gene (chromosome 11) in 143 liver samples. Variant selection was based on dbSNP build 129 and complemented by all variants covered by the HumanHap300v1.1 chip (Illumina) (Figure S1). SNP_ID 65 in the *SLCO1B3_SLCO1B1* genomic region was selected from ref. [78]. Abbreviations: 3'UTR, 3' untranslated region; 5'UTR, 5' untranslated region.

Location of protein residues are based on GenBank reference sequences NP_006437.3 (OATP1B1), NP_062818.1 (OATP1B3), and NP_009187.1 (OATP2B1).

Table S4. Variability of OATP and transcription factor expression in the non-cholestatic liver samples (n=117)

A Variability of *SLCO* mRNA and OATP protein expression

	***SLCO1B1* mRNA**	**OATP1B1 protein**	***SLCO1B3* mRNA**	**OATP1B3 protein**	***SLCO2B1* mRNA**	**OATP2B1 protein**
Minimum	0.906	3.51	0.013	0.53	0.750	2.72
Median	5.681	23.72	0.142	13.05	4.620	13.33
Maximum	24.330	67.22	0.848	45.40	15.006	47.15
Ratio maximum/ minimum	26.9	19.2	65.2	86.5	20.0	17.3
Coefficient of variation (%)	67.3	53.9	75.9	59.1	62.7	48.6

Expression of mRNA and protein was determined by TaqMan technology and immunoblotting, respectively.

B Variability of transcription factor mRNA expression

	HNF1α	***FXR***	***LXRα***	***CAR***	***AhR***	***Sp1***	***HNF3β*[1]**	***HNF3β*[2]**	***HNF3β*[3]**
Minimum	112.2	477.7	1342	704.3	7434	219.8	61.4	50.6	1314
Median	178.5	982.3	2257	1510	11585	337.8	69.1	59.7	2452
Maximum	270.6	1520	3304	2226	17929	564.2	86.2	69.1	5873
Ratio maximum/ minimum	2.4	3.2	2.5	3.2	2.4	2.6	1.4	1.4	4.5
Coefficient of variation (%)	18.4	21.3	16.0	22.7	20.6	15.9	6.0	6.2	28.7

Expression data were extracted from Schröder et al. [27] (GEO dataset GSE32504). The antilogarithms of the expression values to base 2 were calculated and used for the analysis. HNF3β[1]: transcript variant 1 (NM_021784); HNF3β[2]: transcript variant 2 (NM_133675), probe 1; HNF3β[3]: transcript variant 2 (NM_133675), probe 2

Significant positive correlations were detected between expression of *SLCO1B1* and *HNF1α*, *FXR* and *LXRα* expression as well as between *SLCO1B3* and *HNF1α* and *FXR* being in line with previous reports using *in vitro* assays [14,15,17,20,21]. A significant negative correlation was found between *AhR* and *SLCO1B3* and *SLCO2B1* expression as suggested by *in vitro* assays as well [19]. However, contrary to results obtained *in vitro* [16,17,19,21], *Sp1* and *HNF3β* expression were not significantly associated with *SLCO* mRNA expression, and *CAR* expression was positively correlated with *SLCO* expression.

Table S5. Multivariate analysis of hepatic OATP expression in relation to 8 non-genetic factors in the non-cholestatic liver samples (n=117)

	***P* value**					
**Non-genetic factor**	***SLCO1B1* mRNA**	**OATP1B1 protein**	***SLCO1B3* mRNA**	**OATP1B3 protein**	***SLCO2B1* mRNA**	**OATP2B1 protein**
Sex	0.797	0.949	0.635	0.854	0.676	0.280
Age	0.464	0.440	0.592	0.021	0.885	0.432
Smoking habit	0.392	0.549	0.314	0.734	0.190	0.370
Alcohol consumption	0.055	0.141	0.049	0.765	0.001	0.675
Pre-surgery drugs	0.296	0.784	0.667	0.352	0.748	0.189
Diagnosis	0.718	0.164	0.197	0.363	0.708	0.284
Bilirubin	0.512	0.678	0.513	0.571	0.776	0.372
γ-glutamyl transferase	0.329	0.818	0.734	0.298	0.378	0.284

Table S6. Multivariate analysis of hepatic OATP expression in the non-cholestatic liver samples in relation to genetic variants, corrected for non-genetic covariates and regulatory factors considering different genetic models.

SLCO1B3-SLCO1B1 genomic region										
			***SLCO1B1* mRNA**		**OATP1B1 protein**		***SLCO1B3* mRNA**		**OATP1B3 protein**	
**Genetic variant**	**Genetic model**		**Unadjusted *P* value**	**Holm-adjusted *P* value**	**Unadjusted *P* value**	**Holm-adjusted *P* value**	**Unadjusted *P* value**	**Holm-adjusted *P* value**	**Unadjusted *P* value**	**Holm-adjusted *P* value**
rs1588918	Codominant	AA vs. AC vs. CC	0.86	1	0.54	1	0.57	1	0.87	1
	Dominant	AA vs. AC/CC	0.64	1	0.37	1	0.86	1	0.77	1
	Recessive	AA/AC vs. CC	0.69	1	0.70	1	0.34	1	0.62	1
	Additive	0,1,2	0.59	1	0.59	1	0.77	1	0.65	1
rs4762785	Codominant	CC vs. AC vs. AA	0.93	1	0.87	1	0.60	1	0.67	1
	Dominant	CC vs. AC/AA	0.85	1	0.82	1	0.69	1	0.38	1
	Recessive	CC/AC vs. AA	0.71	1	0.71	1	0.31	1	0.67	1
	Additive	0,1,2	0.76	1	0.98	1	0.46	1	0.38	1
rs10841644	Codominant	CC vs. AC vs. AA	0.93	1	0.80	1	0.32	1	0.68	1
	Dominant	CC vs. AC/AA	0.83	1	0.59	1	0.75	1	0.38	1
	Recessive	CC/AC vs. AA	0.81	1	0.79	1	0.17	1	0.85	1
	Additive	0,1,2	0.93	1	0.73	1	0.79	1	0.42	1
rs7978273	Additive (codominant)*	AA vs. AG	0.47	1	0.0029	0.12	0.91	1	0.04	1
rs1356148	Codominant	TT vs. TC vs. CC	0.08	1	0.00018	**0.009**	0.10	1	0.35	1
	Dominant	TT vs. TC/CC	0.46	1	3.12E-05	**0.002**	0.18	1	0.15	1
	Recessive	TT/TC vs. CC	0.054	1	0.52	1	0.17	1	0.90	1
	Additive	0,1,2	0.89	1	0.0019	**0.0096**	0.56	1	0.21	1
rs11045521	Codominant	GG vs. GA vs. AA	0.64	1	0.01	0.37	0.82	1	0.52	1
	Dominant	GG vs. GA/AA	1.00	1	0.0029	0.11	0.75	1	0.25	1
	Recessive	GG/GA vs. AA	0.36	1	0.28	1	0.65	1	0.79	1
	Additive	0,1,2	0.78	1	0.0028	0.11	0.88	1	0.27	1
rs1515766	Additive (codominant)*	TT vs. TC	0.47	1	0.049	1	0.69	1	0.57	1

***SLCO1B3-SLCO1B1* genomic region, continued**										
			***SLCO1B1* mRNA**		**OATP1B1 protein**		***SLCO1B3* mRNA**		**OATP1B3 protein**	
**Genetic variant**	**Genetic model**		**Unadjusted *P* value**	**Holm-adjusted *P* value**	**Unadjusted *P* value**	**Holm-adjusted *P* value**	**Unadjusted *P* value**	**Holm-adjusted *P* value**	**Unadjusted *P* value**	**Holm-adjusted *P* value**
rs10841661	Codominant	GG vs. AG vs. AA	0.38	1	8.53E-06	**0.0005**	0.31	1	0.09	1
	Dominant	GG vs. AG/AA	0.28	1	9.03E-06	**0.0005**	0.18	1	0.03	1
	Recessive	GG/AG vs. AA	0.23	1	0.97	1	0.25	1	0.66	1
	Additive	0,1,2	0.17	1	0.003	0.11	0.12	1	0.09	1
rs7310629	Codominant	GG vs. AG vs. AA	0.74	1	0.008	0.29	0.91	1	0.78	1
	Dominant	GG vs. AG/AA	0.84	1	0.002	0.09	0.84	1	0.48	1
	Recessive	GG/AG vs. AA	0.48	1	0.27	1	0.72	1	0.79	1
	Additive	0,1,2	0.98	1	0.002	0.08	0.94	1	0.48	1
rs7306033	Codominant	AA vs. AG vs. GG	0.27	1	0.0008	**0.04**	0.75	1	0.32	1
	Dominant	AA vs. AG/GG	0.67	1	0.0001	**0.007**	0.65	1	0.13	1
	Recessive	AA/AG vs. GG	0.11	1	0.61	1	0.59	1	0.86	1
	Additive	0,1,2	0.38	1	0.0003	**0.014**	0.81	1	0.15	1
rs7311358 (100% linked with rs2900474, rs4149117, rs3764009)	Codominant	AA vs. GA vs. GG	0.20	1	0.83	1	0.07	1	0.37	1
	Dominant	AA vs. GA/GG	0.07	1	0.73	1	0.02	1	0.22	1
	Recessive	AA/GA vs. GG	0.45	1	0.55	1	0.35	1	0.28	1
	Additive	0,1,2	0.08	1	0.63	1	0.02	1	0.17	1
rs60140950	Codominant	GG vs. GC vs. CC	0.20	1	0.001	0.051	0.73	1	0.31	1
	Dominant	GG vs. GC/CC	0.47	1	0.0002	**0.011**	0.71	1	0.13	1
	Recessive	GG/GC vs. CC	0.08	1	0.62	1	0.53	1	0.86	1
	Additive	0,1,2	0.24	1	0.0004	**0.020**	0.88	1	0.15	1
rs6487172	Codominant	GG vs. AG vs. AA	0.83	1	0.013	0.41	0.32	1	0.15	1
	Dominant	GG vs. AG/AA	0.71	1	0.0037	0.13	0.13	1	0.07	1
	Recessive	GG/AG vs. AA	0.79	1	0.44	1	0.50	1	0.14	1
	Additive	0,1,2	0.90	1	0.017	0.49	0.17	1	0.051	1
rs11045585	Codominant	AA vs. AG vs. GG	0.23	1	0.84	1	0.14	1	0.32	1
	Dominant	AA vs. AG/GG	0.09	1	0.89	1	0.049	1	0.18	1
	Recessive	AA/AG vs. GG	0.45	1	0.55	1	0.35	1	0.28	1
	Additive	0,1,2	0.09	1	0.75	1	0.047	1	0.13	1
rs3764006	Additive (codominant)*	AA vs. AG	0.13	1	0.59	1	0.02	1	0.32	1
rs10841707 (100% linked with rs919842)	Additive (codominant)*	AA vs. AT	0.15	1	0.84	1	0.054	1	0.43	1

***SLCO1B3-SLCO1B1* genomic region, continued**										
			***SLCO1B1* mRNA**		**OATP1B1 protein**		***SLCO1B3* mRNA**		**OATP1B3 protein**	
**Genetic variant**	**Genetic model**		**Unadjusted *P* value**	**Holm-adjusted *P* value**	**Unadjusted *P* value**	**Holm-adjusted *P* value**	**Unadjusted *P* value**	**Holm-adjusted *P* value**	**Unadjusted *P* value**	**Holm-adjusted *P* value**
rs10841712	Codominant	GG vs. AG vs. AA	0.54	1	0.026	0.76	0.40	1	0.71	1
	Dominant	GG vs. AG/AA	0.42	1	0.0096	0.31	0.22	1	0.44	1
	Recessive	GG/AG vs. AA	0.61	1	0.14	1	0.83	1	0.60	1
	Additive	0,1,2	0.71	1	0.008	0.25	0.41	1	0.40	1
rs10841714	Additive (codominant)*	AA vs. AG	0.08	1	0.83	1	0.0086	0.47	0.47	1
rs11519061	Codominant	GG vs. AG vs. AA	0.99	1	0.031	0.83	0.98	1	0.50	1
	Dominant	GG vs. AG/AA	0.92	1	0.015	0.44	0.88	1	0.32	1
	Recessive	GG/AG vs. AA	0.93	1	0.16	1	0.88	1	0.67	1
	Additive	0,1,2	0.95	1	0.008	0.25	0.86	1	0.44	1
rs2417873	Codominant	GG vs. AG vs. AA	0.89	1	0.011	0.37	0.61	1	0.86	1
	Dominant	GG vs. AG/AA	0.63	1	0.0027	0.11	0.43	1	0.75	1
	Recessive	GG/AG vs. AA	0.85	1	0.19	1	0.41	1	0.60	1
	Additive	0,1,2	0.65	1	0.004	0.15	0.33	1	0.64	1
rs10505870	Codominant	GG vs. AG vs. AA	0.39	1	0.91	1	0.42	1	0.68	1
	Dominant	GG vs. AG/AA	0.53	1	0.71	1	0.61	1	0.93	1
	Recessive	GG/AG vs. AA	0.18	1	0.74	1	0.19	1	0.39	1
	Additive	0,1,2	0.26	1	0.66	1	0.31	1	0.64	1
rs1910186	Additive (codominant)*	AA vs. AC	0.09	1	0.40	1	0.10	1	0.003	0.19
rs11045659	Codominant	GG vs. AG vs. AA	0.58	1	0.018	0.53	0.60	1	0.86	1
	Dominant	GG vs. AG/AA	0.98	1	0.009	0.28	0.75	1	0.59	1
	Recessive	GG/AG vs. AA	0.31	1	0.12	1	0.39	1	0.98	1
	Additive	0,1,2	0.72	1	0.004	0.15	0.995	1	0.65	1
rs11045689	Codominant	AA vs. AG vs. GG	0.57	1	0.11	1	0.36	1	0.91	1
	Dominant	AA vs. AG/GG	0.91	1	0.09	1	0.94	1	0.85	1
	Recessive	AA/AG vs. GG	0.33	1	0.08	1	0.18	1	0.67	1
	Additive	0,1,2	0.71	1	0.04	0.98	0.57	1	0.73	1
rs1910169	Additive (codominant)*	GG vs. AG	0.65	1	0.0067	1	0.78	1	0.93	1

SLCO1B3-SLCO1B1 genomic region, continued										
			***SLCO1B1* mRNA**		**OATP1B1 protein**		***SLCO1B3* mRNA**		**OATP1B3 protein**	
**Genetic variant**	**Genetic model**		**Unadjusted *P* value**	**Holm-adjusted *P* value**	**Unadjusted *P* value**	**Holm-adjusted *P* value**	**Unadjusted *P* value**	**Holm-adjusted *P* value**	**Unadjusted *P* value**	**Holm-adjusted *P* value**
rs11045773	Additive (codominant)*	AA vs. AG	0.89	1	0.06	1	0.63	1	0.36	1
rs852550 (100% linked with rs852549)	Additive (codominant)*	AA vs. AG	0.07	1	0.90	1	0.06	1	0.71	1
rs17328763	Additive (codominant)*	TT vs. CT	0.60	1	0.028	0.78	0.26	1	0.51	1
rs4149015	Additive (codominant)*	GG vs. GA	0.58	1	0.16	1	0.40	1	0.45	1
rs3829306 (100% linked with rs4149013)	Additive (codominant)*	GG vs. AG	0.57	1	0.06	1	0.33	1	0.25	1
rs4149026	Codominant	AA vs. AC vs. CC	0.051	1	0.00027	**0.0140**	0.80	1	0.75	1
	Dominant	AA vs. AC/CC	0.80	1	0.00030	**0.0143**	0.71	1	0.55	1
	Recessive	AA/AC vs. CC	0.03	1	0.0043	0.21	0.51	1	0.81	1
	Additive	0,1,2	0.34	1	4.96E-05	**0.0027**	0.55	1	0.78	1
rs4149030	Codominant	GG vs. AG vs. AA	0.06	1	0.0008	**0.037**	0.051	1	0.22	1
	Dominant	GG vs. AG/AA	0.16	1	0.0002	**0.011**	0.09	1	0.13	1
	Recessive	GG/AG vs. AA	0.24	1	0.0231	1	0.37	1	0.16	1
	Additive	0,1,2	0.89	1	0.0004	**0.0196**	0.63	1	0.08	1
rs2291073	Additive (codominant)*	AA vs. AC	0.30	1	0.0018	0.08	0.83	1	0.20	1
rs4149038	Codominant	AA vs. AG vs. GG	0.65	1	0.08	1	0.37	1	0.53	1
	Dominant	AA vs. AG/GG	0.48	1	0.026	0.73	0.23	1	0.28	1
	Recessive	AA/AG vs. GG	0.65	1	0.92	1	0.60	1	0.86	1
	Additive	0,1,2	0.61	1	0.043	1	0.35	1	0.36	1
rs964615	Additive (codominant)*	GG vs. AG	0.60	1	0.51	1	1	1	0.14	1
rs2306283 c.388A>G	Codominant	AA vs. AG vs. GG	0.93	1	1.05E-05	**0.00057**	0.83	1	0.12	1
	Dominant	AA vs. AG/GG	0.77	1	3.10E-06	**0.00017**	0.81	1	0.15	1
	Recessive	AA/AG vs. GG	0.90	1	0.019	0.86	0.65	1	0.06	1
	Additive	0,1,2	0.89	1	6.12E-06	**0.00034**	0.94	1	0.043	1
rs11045818 (100% linked with rs17329885)	Additive (codominant)*	GG vs. AG	0.51	1	0.0004	**0.021**	0.56	1	0.32	1
rs11045819 c.463C>A	Additive (codominant)*	CC vs. CA	0.63	1	0.0036	0.14	0.72	1	0.36	1
rs4149056 c.512T>C	Additive (codominant)*	TT vs. TC	0.09	1	0.19	1	0.10	1	0.42	1

SLCO1B3-SLCO1B1 genomic region, continued										
			***SLCO1B1* mRNA**		**OATP1B1 protein**		***SLCO1B3* mRNA**		**OATP1B3 protein**	
**Genetic variant**	**Genetic model**		**Unadjusted *P* value**	**Holm-adjusted *P* value**	**Unadjusted *P* value**	**Holm-adjusted *P* value**	**Unadjusted *P* value**	**Holm-adjusted *P* value**	**Unadjusted *P* value**	**Holm-adjusted *P* value**
rs4149057	Codominant	CC vs. CT vs. TT	0.64	1	0.39	1	0.65	1	0.18	1
	Dominant	CC vs. CT/TT	0.51	1	0.17	1	0.74	1	0.19	1
	Recessive	CC/CT vs. TT	0.38	1	0.65	1	0.35	1	0.08	1
	Additive	0,1,2	0.37	1	0.25	1	0.48	1	0.08	1
rs2291075	Codominant	CC vs. CT vs. TT	0.44	1	0.00015	**0.0082**	0.53	1	0.44	1
	Dominant	CC vs. CT/TT	0.46	1	3.21E-05	**0.0017**	0.30	1	0.20	1
	Recessive	CC/CT vs. TT	0.45	1	0.07	1	0.94	1	0.66	1
	Additive	0,1,2	0.93	1	0.00014	**0.0075**	0.51	1	0.27	1
rs2100996	Codominant	AA vs. AG vs. GG	0.90	1	0.00045	**0.022**	0.98	1	0.11	1
	Dominant	AA vs. AG/GG	0.76	1	0.09	1	0.83	1	0.046	1
	Recessive	AA/AG vs. GG	0.67	1	8.64E-05	**0.005**	0.97	1	0.16	1
	Additive	0,1,2	0.66	1	0.001	**0.046**	0.87	1	0.037	1
rs11045834	Codominant	GG vs. AG vs. AA	0.87	1	0.0047	0.18	0.12	1	0.07	1
	Dominant	GG vs. AG/AA	0.70	1	0.0016	0.07	0.51	1	0.026	1
	Recessive	GG/AG vs. AA	0.66	1	0.11	1	0.041	1	0.97	1
	Additive	0,1,2	0.62	1	0.0011	**0.048**	0.16	1	0.07	1
rs4149064	Additive (codominant)*	AA vs. AG	0.97	1	0.56	1	0.10	1	0.40	1
rs4363657	Additive (codominant)*	AA vs. AG	0.37	1	0.07	1	0.22	1	0.51	1
rs987839	Codominant	AA vs. AG vs. GG	0.98	1	0.0017	0.08	0.96	1	0.20	1
	Dominant	AA vs. AG/GG	0.87	1	0.07	1	0.95	1	0.09	1
	Recessive	AA/AG vs. GG	0.86	1	0.0004	**0.023**	0.79	1	0.24	1
	Additive	0,1,2	0.84	1	0.0011	**0.047**	0.84	1	0.08	1
rs7966613	Codominant	AA vs. AG vs. GG	0.42	1	0.07	1	0.75	1	0.41	1
	Dominant	AA vs. AG/GG	0.32	1	0.024	0.70	0.45	1	0.19	1
	Recessive	AA/AG vs. GG	0.66	1	0.65	1	0.88	1	0.78	1
	Additive	0,1,2	0.62	1	0.07	1	0.54	1	0.28	1
rs10841763	Codominant	AA vs. AG vs. GG	0.38	1	0.10	1	0.61	1	0.40	1
	Dominant	AA vs. AG/GG	0.30	1	0.033	0.85	0.40	1	0.23	1
	Recessive	AA/AG vs. GG	0.64	1	0.53	1	0.86	1	0.89	1
	Additive	0,1,2	0.60	1	0.07	1	0.60	1	0.43	1

***SLCO1B3-SLCO1B1* genomic region, continued**										
			***SLCO1B1* mRNA**		**OATP1B1 protein**		***SLCO1B3* mRNA**		**OATP1B3 protein**	
**Genetic variant**	**Genetic model**		**Unadjusted *P* value**	**Holm-adjusted *P* value**	**Unadjusted *P* value**	**Holm-adjusted *P* value**	**Unadjusted *P* value**	**Holm-adjusted *P* value**	**Unadjusted *P* value**	**Holm-adjusted *P* value**
rs10841767	Codominant	AA vs. AG vs. GG	0.31	1	0.00078	**0.037**	0.31	1	0.24	1
	Dominant	AA vs. AG/GG	0.79	1	0.00018	**0.0089**	0.79	1	0.15	1
	Recessive	AA/AG vs. GG	0.12	1	0.22	1	0.13	1	0.56	1
	Additive	0,1,2	0.52	1	0.00016	**0.0081**	0.52	1	0.23	1
rs34671512 c.1929A>C	Additive (codominant)*	AA vs. CA	0.51	1	0.011	0.37	0.94	1	0.45	1
rs11045893 (100% linked with rs11045891, rs11045892)	Codominant	TT vs. TC vs. CC	0.35	1	0.0023	0.098	0.33	1	0.22	1
	Dominant	TT vs. TC/CC	0.64	1	0.0006	**0.027**	0.81	1	0.13	1
	Recessive	TT/TC vs. CC	0.15	1	0.21	1	0.14	1	0.56	1
	Additive	0,1,2	0.41	1	0.0005	**0.022**	0.54	1	0.21	1
rs12372157 (100% linked with rs4149087)	Codominant	TT vs. GT vs. GG	0.73	1	0.33	1	0.76	1	0.43	1
	Dominant	TT vs. GT/GG	0.64	1	0.14	1	0.73	1	0.2	1
	Recessive	TT/GT vs. GG	0.67	1	0.42	1	0.62	1	0.47	1
	Additive	0,1,2	0.91	1	0.15	1	0.99	1	0.21	1
rs9804732	Codominant	CC vs. AC vs. AA	0.85	1	0.34	1	0.76	1	0.41	1
	Dominant	CC vs. AC/AA	0.68	1	0.15	1	0.75	1	0.21	1
	Recessive	CC/AC vs. AA	0.80	1	0.48	1	0.60	1	0.40	1
	Additive	0,1,2	0.87	1	0.17	1	0.96	1	0.19	1
rs7975087	Codominant	AA vs. AC vs. CC	0.41	1	0.016	0.50	0.18	1	0.24	1
	Dominant	AA vs. AC/CC	0.30	1	0.0045	0.16	0.20	1	0.14	1
	Recessive	AA/AC vs. CC	0.31	1	0.29	1	0.12	1	0.25	1
	Additive	0,1,2	0.20	1	0.0046	0.15	0.09	1	0.09	1
rs7311964	Codominant	AA vs. AC vs. CC	0.25	1	0.007	0.26	0.06	1	0.37	1
	Dominant	AA vs. AC/CC	0.30	1	0.003	0.11	0.18	1	0.16	1
	Recessive	AA/AC vs. CC	0.13	1	0.12	1	0.028	1	0.60	1
	Additive	0,1,2	0.14	1	0.002	0.07	0.044	1	0.17	1

SLCO2B1						
			***SLCO2B1* mRNA**		**OATP2B1 protein**	
**Genetic variant**	**Genetic model**		**Unadjusted *P* value**	**Holm-adjusted *P* value**	**Unadjusted *P* value**	**Holm-adjusted *P* value**
rs4944989	Additive (codominant)*	GG vs. AG	0.11	1	0.93	1
rs4944991	Codominant	TT vs. CT vs. CC	0.09	0.94	0.69	1
	Dominant	TT vs. CT/CC	0.08	0.92	0.51	1
	Recessive	TT/CT vs. CC	0.07	0.76	0.76	1
	Additive	0,1,2	0.04	0.42	0.65	1
rs2851067 (100% linked with rs2851069)	Codominant	GG vs. GT vs. TT	0.93	1	0.66	1
	Dominant	GG vs. GT/TT	0.88	1	0.37	1
	Recessive	GG/GT vs. TT	0.77	1	0.92	1
	Additive	0,1,2	0.97	1	0.48	1
rs4100076	Codominant	AA vs. CA vs. CC	0.07	0.85	0.49	1
	Dominant	AA vs. CA/CC	0.07	0.90	0.43	1
	Recessive	AA/CA vs. CC	0.06	0.76	0.51	1
	Additive	0,1,2	0.03	0.40	0.63	1
rs2712807	Additive (codominant)*	AA vs. GA	0.02	0.21	0.77	1
rs4944994	Codominant	AA vs. AG vs. GG	0.18	1	0.58	1
	Dominant	AA vs. AG/GG	0.11	1	0.34	1
	Recessive	AA/AG vs. GG	0.16	1,00	0.94	1
	Additive	0,1,2	0.07	0.70	0.45	1
rs1789694	Codominant	AA vs. AG vs. GG	0.38	1	0.21	1
	Dominant	AA vs. AG/GG	0.27	1	0.76	1
	Recessive	AA/AG vs. GG	0.50	1	0.08	0.99
	Additive	0,1,2	0.42	1	0.45	1
rs12422149	Additive (codominant)*	GG vs. GA	0.22	1	0.35	1
rs10501421	Additive (codominant)*	GG vs. GA	0.69	1	0.90	1
rs2306168 (100% linked with rs3824904, rs765824)	Additive (codominant)*	CC vs. CT	0.15	1	0.33	1
rs7116044	Additive (codominant)*	CC vs. AC	0.64	1	0.56	1
rs17133815	Additive (codominant)*	GG vs. AG	0.81	1	0.85	1
rs7924924	Additive (codominant)*	AA vs. AG	0.96	1	0.76	1

Four different genetic models were considered: codominant (homozygote allele 1 vs. heterozygote vs. homozygote allele 2), dominant (homozygote allele 1 vs. heterozygote + homozygote allele 2), recessive (homozygote allele 1 + heterozygote vs. homozygote allele 2) and additive (homozygote allele 1, heterozygote and homozygote allele 2 coded numerically as 0, 1, 2). The effects were corrected for 8 non-genetic factors (sex, age, smoking habit, alcohol consumption, pre-surgery drugs, diagnosis, bilirubin, γ-glutamyl transferase) and 7 transcription factors (HNF1α, Sp1, AhR, CAR, FXR, LXRα, HNF3β).

The four *SLCO1B1* missense variants present in the liver cohort (c.388A>G, rs2306283; c.463C>A, rs11045819; c.521T>C, rs4149056; c.1929A>C, rs34671512) used for calculating haplotypes (Fig. 2C) and also previously described as key variants [8,80] are indicated in orange.

In case of 100% linkage between variants we only included one variant in the analysis.

*Genetic variants without homozygote carriers of allele 2 (i.e. codominant model or additive model).

Holm-adjusted *P* values <0.05 are given in boldface.

Table S7. *SLCO1B1* haplotype frequencies and their effects on OATP1B1 expression.

SLCO1B1 haplotypes and their effects on *SLCO1B1* mRNA expression

Haplotype	rs11045773	rs852550	rs852549	rs4149013	rs17328763	rs4149015	rs3829306	rs4149026	rs4149030	rs2291073	rs4149038	rs17329885	rs964615	rs2306283	rs11045818	rs11045819	rs4149056	rs4149057	rs2291075	rs2100996	rs11045834	rs4149064	rs4363657	rs987839	rs7966613	rs10841763	rs10841767	rs34671512	rs4149087	rs11045891	rs11045892	rs11045893	rs12372157	rs9804732	rs7975087	rs7311964	Frequency (%)	Effect size	Unadjusted *P* value	Holm-adjusted *P* value
Reference H-1B1_01																																					33.0	-	-	-
H-1B1_02																																					17.6	-0.07	0.444	1
H-1B1_03																																					5.3	0.30	**0.0278**	0.167
H-1B1_04																																					5.0	-0.15	0.291	1
H-1B1_05																																					3.5	-0.03	0.857	1
H-1B1_06																																					3.1	-0.08	0.631	1

SLCO1B1 haplotypes and their effects on OATP1B1 protein expression

Haplotype	rs11045773	rs852550	rs852549	rs4149013	rs17328763	rs4149015	rs3829306	rs4149026	rs4149030	rs2291073	rs4149038	rs17329885	rs964615	rs2306283	rs11045818	rs11045819	rs4149056	rs4149057	rs2291075	rs2100996	rs11045834	rs4149064	rs4363657	rs987839	rs7966613	rs10841763	rs10841767	rs34671512	rs4149087	rs11045891	rs11045892	rs11045893	rs12372157	rs9804732	rs7975087	rs7311964	Frequency (%)	Effect size	Unadjusted *P* value	Holm-adjusted *P* value
Reference H-1B1_01																																					34.0	-	-	-
H-1B1_02																																					17.5	0.51	**0.0002**	**0.0012**
H-1B1_03																																					5.8	0.11	0.571	1
H-1B1_04																																					3.8	0.11	0.599	1
H-1B1_06																																					3.4	0.76	**0.0013**	**0.0078**
H-1B1_05																																					3.2	0.05	0.837	1
H-1B1_07																																					2.0	-0.29	0.301	1

SLCO1B1 haplotype frequencies and their effects on OATP1B1 expression were corrected for 8 non-genetic factors (see Supplementary Table S5) and 7 transcription factors (HNF1α, Sp1, AhR, CAR, FXR, LXRα, HNF3β). Haplotypes were calculated for all variants listed in Supplementary Table S3 with a frequency ≥1%. Effect sizes and *P* values are given for haplotypes with frequencies ≥2%. Differences in haplotype frequencies between both tables are due to missing protein data in 11 cases. The variants rs2306283 (c.388A>G) and rs4149056 (c.521T>C) are indicated in orange.

Table S8. *SLCO1B3* haplotype frequencies and their effects on OATP1B3 expression.

SLCO1B3 haplotypes and their effects on *SLCO1B3* mRNA expression

Haplotype	rs1588918	rs4762785	rs10841644	rs7978273	rs1356148	rs11045521	rs1515766	rs10841661	rs7310629	rs2900474	rs4149117	rs3764009	rs7306033	rs7311358	rs60140950	rs6487172	rs11045585	rs3764006	rs10841707	rs919842	rs10841712	rs10841714	rs11519061	rs2417873	Frequency (%)	Effect size	Unadjusted *P* value	Holm-adjusted *P* value
Reference H-1B3_01																									34.1	-	-	-
H-1B3_02																									11.5	0.04	0.257	1
H-1B3_03																									4.6	0.04	0.513	1
H-1B3_04																									4.4	0.05	0.348	1
H-1B3_05																									4.0	0.10	**0.0374**	0.336
H-1B3_06																									3.2	-0.02	0.701	1
H-1B3_07																									2.7	0.01	0.915	1
H-1B3_08																									2.4	0.18	**0.0077**	0.077
H-1B3_09																									2.3	0.01	0.898	1
H-1B3_10																									2.2	0.07	0.294	1

SLCO1B3 haplotypes and their effects on OATP1B3 protein expression

Haplotype	rs1588918	rs4762785	rs10841644	rs7978273	rs1356148	rs11045521	rs1515766	rs10841661	rs7310629	rs2900474	rs4149117	rs3764009	rs7306033	rs7311358	rs60140950	rs6487172	rs11045585	rs3764006	rs10841707	rs919842	rs10841712	rs10841714	rs11519061	rs2417873	Frequency (%)	Effect size	Unadjusted *P* value
Reference H-1B3_01																									33.3	-	-
H-1B3_02																									11.2	0.03	0.794
H-1B3_04																									5.1	0.26	0.202
H-1B3_05																									4.4	0.09	0.581
H-1B3_03																									3.9	0.06	0.760
H-1B3_06																									3.0	-0.33	0.109
H-1B3_08																									3.0	-0.37	0.094
H-1B3_07																									3.0	0.35	0.086
H-1B3_09																									2.5	-0.33	0.159
H-1B3_11																									2.2	0.29	0.225

SLCO1B3 haplotype frequencies and their effects on OATP1B3 expression were corrected for 8 non-genetic factors (see Supplementary Table S5) and 7 transcription factors (HNF1α, Sp1, AhR, CAR, FXR, LXRα, HNF3β). Haplotypes were calculated for all variants listed in Supplementary Table S3 with a frequency ≥1%. Effect sizes and *P* values are given for haplotypes with frequencies ≥2%. Differences in haplotype frequencies between both tables are due to missing protein data in 11 cases.

Table S9. *SLCO2B1* haplotype frequencies and their effects on OATP2B1 expression.

***SLCO2B1* haplotypes and their effects on *SLCO2B1* mRNA expression**

Haplotype	rs4944989	rs4944991	rs2851067	rs4100076	rs2712807	rs2851069	rs4944994	rs1789694	rs12422149	rs10501421	rs765824	rs3824904	rs2306168	rs7116044	rs17133815	rs7924924	Frequency (%)	Effect size	Unadjusted *P* value	Holm-adjusted *P* value
Reference H-2B1_01																	48.2	-	-	-
H-2B1_02			■		■	■		■									17.9	-0.07	0.383	1
H-2B1_03		■	■	■		■	■			■							11.9	0.14	0.079	0.395
H-2B1_04								■									4.1	0.50	**0.0001**	**0.0008**
H-2B1_05		■	■	■		■	■		■								3.8	0.21	0.111	0.444
H-2B1_06														■			3.2	0.10	0.430	1

***SLCO2B1* haplotypes and their effects on OATP2B1 protein expression**

Haplotype	rs4944989	rs4944991	rs2851067	rs4100076	rs2712807	rs2851069	rs4944994	rs1789694	rs12422149	rs10501421	rs765824	rs3824904	rs2306168	rs7116044	rs17133815	rs7924924	Frequency (%)	Effect size	Unadjusted *P* value	Holm-adjusted *P* value
Reference H-2B1_01																	48.4	-	-	-
H-2B1_02			■		■	■		■									17.7	-0.14	0.169	1
H-2B1_03		■	■	■		■	■			■							12.4	-0.04	0.678	1
H-2B1_05		■	■	■		■	■		■								4.0	-0.33	**0.034**	0.236
H-2B1_04								■									3.9	-0.21	0.171	1
H-2B1_06														■			3.9	-0.11	0.451	1
H-2B1_07	■	■	■			■	■		■								2.0	-0.09	0.645	1

SLCO2B1 haplotype frequencies and their effects on OATP2B1 expression were corrected for the 8 non-genetic factors (see Supplementary Table S5) and 7 transcription factors (HNF1α, Sp1, AhR, CAR, FXR, LXRα, HNF3β). Haplotypes were calculated for all variants listed in Supplementary Table S3 with a frequency ≥1%. Effect sizes and *P* values are given for haplotypes with frequencies ≥2%. Differences in haplotype frequencies between both tables are due to missing protein data in 11 cases.

Table S10. Haplotype frequencies of the *SLCO1B3-SLCO1B1* genomic region and effects on expression.

Effects on *SLCO1B1* mRNA expression

					SLCO1B3																				SLCO1B7								SLCO1B1																																				
Haplotype	rs1588918	rs4762785	rs10841644	rs7978273	rs1356148	rs11045521	rs1515766	rs10841661	rs7310629	rs2900474	rs4149117	rs3764009	rs7306033	rs7311358	rs60140950	rs6487172	rs11045585	rs3764006	rs10841707	rs919842	rs10841712	rs10841714	rs11519061	rs2417873	rs10505870	rs1910186	rs11045659	rs11045689	rs1910169	rs11045773	rs852550	rs852549	rs4149013	rs17328763	rs4149015	rs3829306	rs4149026	rs4149030	rs2291073	rs4149038	rs17329885	rs964615	rs2306283	rs11045818	rs11045819	rs4149056	rs4149057	rs2291075	rs2100996	rs11045834	rs4149064	rs4363657	rs987839	rs7966613	rs10841763	rs10841767	rs34671512	rs4149087	rs11045891	rs11045892	rs11045893	rs12372157	rs9804732	rs7975087	rs7311964	Frequency (%)	Effect size	Unadjusted *P* value
Reference H-cluster_01																																																																		17.6	-	-
H-cluster_02																																																																		4.0	0.08	0.619
H-cluster_03																																																																		3.6	0.36	**0.034**
H-cluster_04																																																																		3.4	-0.19	0.268
H-cluster_05																																																																		3.0	-0.05	0.805
H-cluster_06																																																																		2.8	0.31	0.109
H-cluster_07																																																																		2.6	0.04	0.829
H-cluster_08																																																																		2.5	0.14	0.475
H-cluster_09																																																																		2.3	0.23	0.273
H-cluster_10																																																																		2.3	-0.09	0.666
H-cluster_11																																																																		2.2	-0.06	0.786

Effects on OATP1B1 protein expression

					SLCO1B3																				SLCO1B7								SLCO1B1																																				
Haplotype	rs1588918	rs4762785	rs10841644	rs7978273	rs1356148	rs11045521	rs1515766	rs10841661	rs7310629	rs2900474	rs4149117	rs3764009	rs7306033	rs7311358	rs60140950	rs6487172	rs11045585	rs3764006	rs10841707	rs919842	rs10841712	rs10841714	rs11519061	rs2417873	rs10505870	rs1910186	rs11045659	rs11045689	rs1910169	rs11045773	rs852550	rs852549	rs4149013	rs17328763	rs4149015	rs3829306	rs4149026	rs4149030	rs2291073	rs4149038	rs17329885	rs964615	rs2306283	rs11045818	rs11045819	rs4149056	rs4149057	rs2291075	rs2100996	rs11045834	rs4149064	rs4363657	rs987839	rs7966613	rs10841763	rs10841767	rs34671512	rs4149087	rs11045891	rs11045892	rs11045893	rs12372157	rs9804732	rs7975087	rs7311964	Frequency (%)	Effect size	Unadjusted *P* value
Reference H-cluster_01																																																																		20.6	-	-
H-cluster_02																																																																		4.3	0.14	0.543
H-cluster_04																																																																		4.2	0.62	**0.011**
H-cluster_03																																																																		4.0	0.13	0.594
H-cluster_07																																																																		3.0	0	0.991
H-cluster_12																																																																		2.8	0.59	**0.049**
H-cluster_09																																																																		2.0	0.08	0.823
H-cluster_13																																																																		2.0	0.02	0.955

Effects on *SLCO1B3* mRNA expression

					SLCO1B3																				SLCO1B7								SLCO1B1																																				
Haplotype	rs1588918	rs4762785	rs10841644	rs7978273	rs1356148	rs11045521	rs1515766	rs10841661	rs7310629	rs2900474	rs4149117	rs3764009	rs7306033	rs7311358	rs60140950	rs6487172	rs11045585	rs3764006	rs10841707	rs919842	rs10841712	rs10841714	rs11519061	rs2417873	rs10505870	rs1910186	rs11045659	rs11045689	rs1910169	rs11045773	rs852550	rs852549	rs4149013	rs17328763	rs4149015	rs3829306	rs4149026	rs4149030	rs2291073	rs4149038	rs17329885	rs964615	rs2306283	rs11045818	rs11045819	rs4149056	rs4149057	rs2291075	rs2100996	rs11045834	rs4149064	rs4363657	rs987839	rs7966613	rs10841763	rs10841767	rs34671512	rs4149087	rs11045891	rs11045892	rs11045893	rs12372157	rs9804732	rs7975087	rs7311964	Frequency (%)	Effect size	Unadjusted *P* value
Reference H-cluster_01																																																																		17.6	-	-
H-cluster_02																																																																			4.0	0.04	0.457
H-cluster_03																																																																		3.6	0.12	**0.034**
H-cluster_04																																																																		3.4	-0.04	0.519
H-cluster_05																																																																		3.0	-0.04	0.535
H-cluster_06																																																																		2.8	0.01	0.895
H-cluster_07																																																																		2.6	-0.02	0.772
H-cluster_08																																																																		2.5	0.08	0.223
H-cluster_09																																																																		2.3	-0.12	0.086
H-cluster_10																																																																		2.3	0.03	0.676
H-cluster_11																																																																		2.2	0	0.993

Table S10. Haplotype frequencies of the *SLCO1B3-SLCO1B1* genomic region and effects on expression, continued

Effects on OATP1B3 protein expression

					SLCO1B3																				SLCO1B7								SLCO1B1																																			
Haplotype	rs1588918	rs4762785	rs10841644	rs7978273	rs1356148	rs11045521	rs1515766	rs10841661	rs7310629	rs2900474	rs4149117	rs3764009	rs7306033	rs7311358	rs60140950	rs6487172	rs11045585	rs3764006	rs10841707	rs919842	rs10841712	rs10841714	rs11519061	rs2417873	rs10505870	rs1910186	rs11045659	rs11045689	rs1910169	rs11045773	rs852550	rs852549	rs4149013	rs17328763	rs4149015	rs3829306	rs4149026	rs4149030	rs2291073	rs4149038	rs17329885	rs964615	rs2306283	rs11045818	rs11045819	rs4149056	rs4149057	rs2291075	rs2100996	rs11045834	rs4149064	rs4363657	rs987839	rs7966613	rs10841763	rs10841767	rs34671512	rs4149087	rs11045891	rs11045892	rs11045893	rs12372157	rs9804732	rs7975087	rs7311964	Frequency (%)	Effect size	Unadjusted *P* value
Reference H-cluster_01																																																																		20.5	-	-
H-cluster_02																																																																		4.3	0.24	0.183
H-cluster_04																																																																		4.2	0.32	0.110
H-cluster_03																																																																		4.0	0.19	0.318
H-cluster_07																																																																		3.0	0.30	0.157
H-cluster_14																																																																		2.8	0.57	**0.008**
H-cluster_09																																																																		2.0	0.01	0.968
H-cluster_13																																																																		2.0	-0.03	0.906

Haplotype frequencies of the *SLCO1B3-SLCO1B1* genomic region and effects on expression were corrected for 8 non-genetic factors (see Supplementary Table S5) and 7 transcription factors (HNF1α, Sp1, AhR, CAR, FXR, LXRα, HNF3β). Haplotypes were calculated for all variants listed in Supplementary Table S3 with a frequency ≥1%. Effect sizes and *P* values are given for haplotypes with frequencies ≥2%. Differences in haplotype frequencies between tables "*SLCO1B1* mRNA and OATP1B1 protein" or "*SLCO1B3* mRNA and OATP1B3 protein" are due to missing protein data in 11 cases. The variants rs2306283 (c.388A>G) and rs4149056 (c.521T>C) are indicated in orange.

Table S11. *SLCO1B1* haplotypes and atorvastatin pharmacokinetics.

***SLCO1B1* Haplotype**	**Atorvastatin C_{max}**	***P* value**
*1b	1% decrease	0.9615
*4	1% decrease	0.9806
*14	15% decrease	0.3980
*15	33% increase	**0.0310**
*22	21% decrease	0.5639
*35	9% increase	0.7063

***SLCO1B1* Haplotype**	**Atorvastatin half-life**	***P* value**
*1b	19% decrease	**0.0170**
*4	25% decrease	0.1487
*14	2% decrease	0.8529
*15	6% decrease	0.6511
*22	4% increase	0.8072
*35	10% decrease	0.2250

SLCO1B1 haplotypes were calculated based on the 4 missense variants present in the liver cohort as described in legend to Figure 2. Haplotype **15* affects atorvastatin C_{max} and haplotype **1b* atorvastatin half-life. Changes in C_{max} and half-life were calculated in comparison with the reference haplotype **1a*.

Table S12. Kinetic parameters for atorvastatin, rosuvastatin, and estrone sulfate uptake by OATP2B1.

	**OATP2B1-reference**	**OATP2B1-c.601G>A**	**OATP2B1-c.935G>A**	**OATP2B1-c.1457C>T**
**Atorvastatin**				
K_m	2.2 ± 0.5	2.4 ± 0.6	3.2 ± 0.6 #	2.1 ± 0.8
V_{max}	17.4 ± 3.5	20.0 ± 3.1	20.7 ± 3.7	13.2 ± 2.9 §
V_{max}/K_m	8.3 ± 0.5	9.0 ± 1.2	6.6 ± 0.2	7.8 ± 1.9
**Rosuvastatin**				
K_m	4.1 ± 0.1	5.5 ± 1.1	6.4 ± 0.5	3.6 ± 0.5
V_{max}	46.6 ± 4.7	49.7 ± 10.8	51.5 ± 0.9	34.7 ± 3.3
V_{max}/K_m	11.3 ± 1.2	9.0 ± 0.5	8.1 ± 0.6	9.9 ± 0.6
**Estrone sulfate**				
K_m	16.9 ± 1.3	21.3 ± 3.6	17.8 ± 6.5	22.3 ±11.8
V_{max}	1148 ± 35	1748 ± 325	751 ± 132	879 ± 262
V_{max}/K_m	68.9 ± 6.2	81.7 ± 1.7	48.5 ± 8.5 ¶	49.1 ± 9.4 ¶

The kinetic parameters K_m (µmol/L) and V_{max} (pmol • mg protein^{-1} • min^{-1}) were calculated from data shown in Supplementary Figure S4. V_{max} values were normalized by the expression level of OATP2B1-reference. Values are means ± SE. Significance was tested using repeated measures ANOVA with Tukey's multiple comparison test as post test.

# P<0.05 vs. reference, c.601G>A, and c.1457C>T

§ P<0.05 vs. c.601G>A and c.935G>A

¶ P<0.05 vs. reference and c.601G>A

Table S13. *In silico* prediction of functional effects of OATP2B1 missense variants.

Variant*	Amino acid change**	SIFT (score)	PolyPhen-2 (score)	PMut (score; reliability)	SNPs3D (score)
c.601G>A	Val201Met	Tolerated (0.16)	Probably damaging (0.983)	Neutral (0.2212; 5)	Non-deleterious (0.62)
c.935G>A	Arg312Gln	Tolerated (0.58)	Benign (0.016)	Neutral (0.4488; 1)	Non-deleterious (0.65)
c.1457C>T	Ser486Phe	Tolerated (0.71)	Benign (0.000)	Neutral (0.4337; 1)	Non-deleterious (2.48)

Functional effects of OATP2B1 missense variants were predicted with SIFT (Sorting Intolerant from Tolerant) Human Protein DB tool [67,68], PolyPhen-2 [65,66], PMut [69,70], and SNPs3D [71,72]. SIFT scores range from 0 to 1. The amino acid substitution is predicted “damaging” if the score is ≤0.05, and “tolerated” if the score is >0.05. PolyPhen-2 scores also range from 0 to 1. Here, variants with a high score (>0.85) are considered as being “probably damaging”, variants with a score between 0.15 and 0.85 as being “possibly damaging”, and variants with a score <0.15 as “benign”. PMut scores of 0-0.5 are designated “neutral” and those of 0.5-1 “pathological”. Reliability ranges from 0-9, and scores >5 are considered reliable. The SNPs3D algorithm classifies variants as “deleterious” when scores are <0 and as “non-deleterious” when scores are >0.

*in relation to NM_007256.4

**in relation to NP_009187.1

Table S14. *SLCO* genetic variants affect expression of hepatic OATPs in the total sample set.

SLCO1B1 haplotypes, calculated based on the four missense variants present in the liver cohort (c.388A>G, c.463C>A, c.521T>C, c.1929A>C) and also previously described as key variants [8,80], affect OATP1B1 protein expression. Only haplotypes with frequencies ≥2% are given. The effect sizes indicate differences of OATP1B1 expression compared to reference haplotype *SLCO1B1*1a*. The analysis was performed in the total sample set including the non-cholestatic and the cholestatic liver samples. Boldface: significant *P* values.

	**c.388A>G**	**c.463C>A**	**c.521T>C**	**c.1929A>C**	**Frequency (%)**	**Effect size**	**Unadjusted *P* value**	**Holm-adjusted *P* value**
*1a					51.9	-	-	-
*1b					8.5	0.42	**0.0027**	**0.0082**
*5					3.4	0.01	0.9544	1
*14					16.3	0.50	**1.2×10^{-5}**	**6.1×10^{-5}**
*15					13.1	0.05	0.6076	1
*35					6.2	0.58	**0.0002**	**0.0006**

Table S15. Sequences of oligonucleotide primers and TaqMan probes.

Gene	NCBI accession number	Oligonucleotide sequence	Exon location	Final conc.
SLCO1B1	NM_006446.4			
Sense primer		5’-CAACAGTATGGTCAGCCTTCATCT-3’	9	400 nM
Antisense primer		5’-TTCCACTTGCAAAAATAGGTATGG-3’	10	400 nM
Probe		5’-FAM-CTAACATCTTATTGGGAGTC-3’-MGB	9/10	200 nM
SLCO1B3	NM_019844.2			
Sense primer		5’-CCAACAGCTGTGGAGCACAA-3’	14	300 nM
Antisense primer		5’-AGTGCTGGGAATCTTAAAGCTATAGATAA-3’	15	300 nM
Probe		5’-FAM-CCAAGTAGACCCTTCC-3’-MGB	14/15	200 nM
SLCO2B1	NM_007256.4			
Sense primer		5’-TCATGCTGCGCCTTTATGTG-3’	7	400 nM
Antisense primer		5’-CGGCAGCGATGAGGAAAC-3’	8	400 nM
Probe		5’-FAM-CCACCTTCTGGCATC-3’-MGB	7/8	200 nM

FAM, 6-carboxyfluorescein; MGB, minor groove binder

Neither primers nor probes covered a described genetic variant (NCBI SNP build 132).

Each *SLCO* TaqMan assay was specific for the respective *SLCO* mRNA and did not detect the other 2 *SLCO* mRNAs.

Figure S1. Selection of *SLCO* genetic variants for genotyping, genotyping methods, variants detected and used for statistical analysis. Matrix-assisted laser desorption/ionization time-of-flight mass spectrometry (MALDI-TOF MS) using the MassARRAY Compact system (Sequenom, San Diego, CA) was performed as described [79]. Chip analysis was performed at the Microarray Facility of the University of Tübingen, Germany, as described [27].

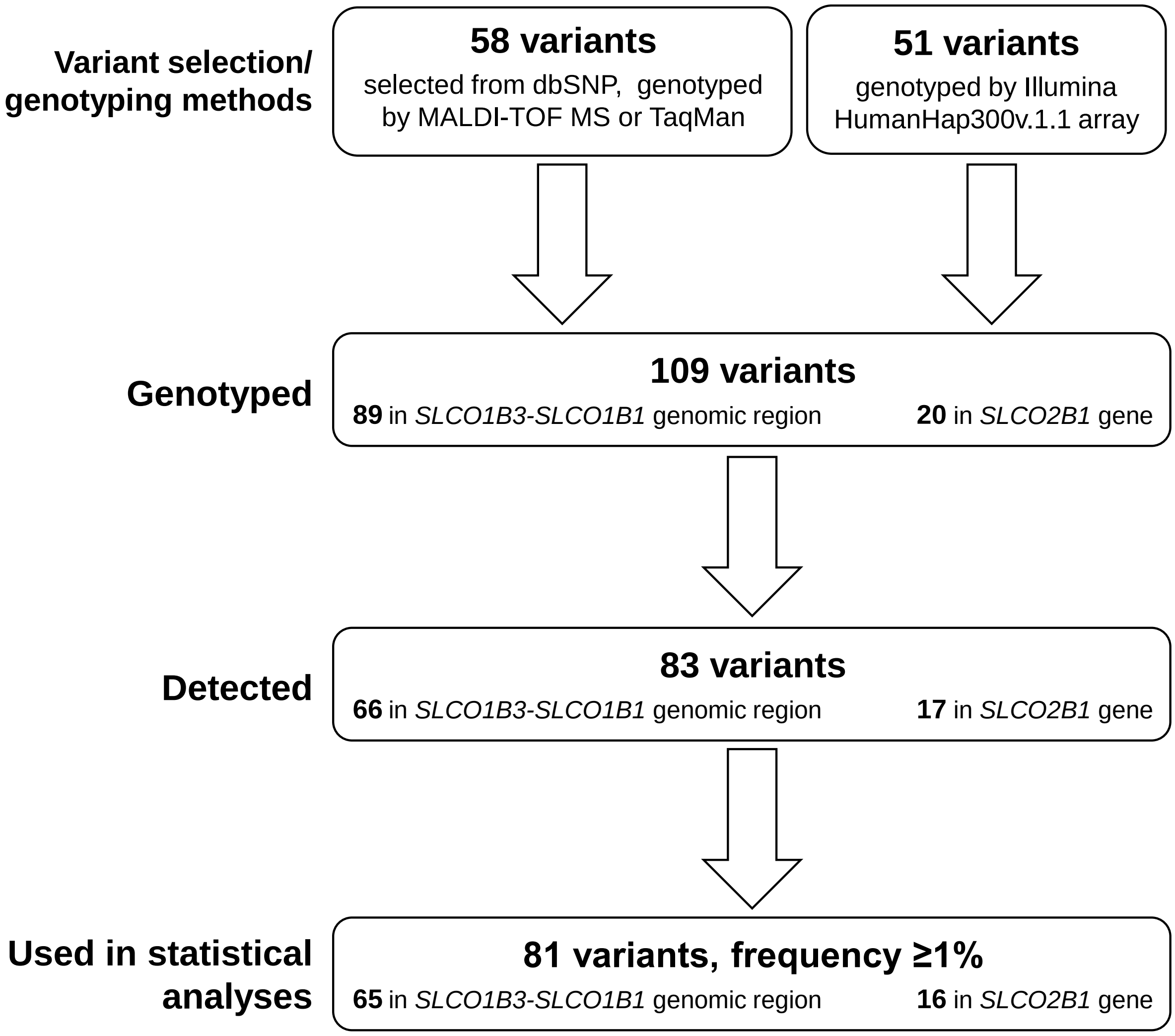

Figure S2. Systematic analysis of OATP expression in human liver samples. (A,C,E) *SLCO1B1* mRNA (NM_006446.4), *SLCO1B3* mRNA (NM_019844.2), and *SLCO2B1* mRNA (NM_007256.4) were quantified by TaqMan technology and normalized to *β-actin* mRNA levels. (B,D,F) OATP protein expression was analyzed by immunoblotting using previously characterized highly specific antibodies [28,29]. The panels show histograms in combination with probability plots for mRNA and protein quantification from 143 and 132 human liver samples, respectively. Expression data were not normally distributed.

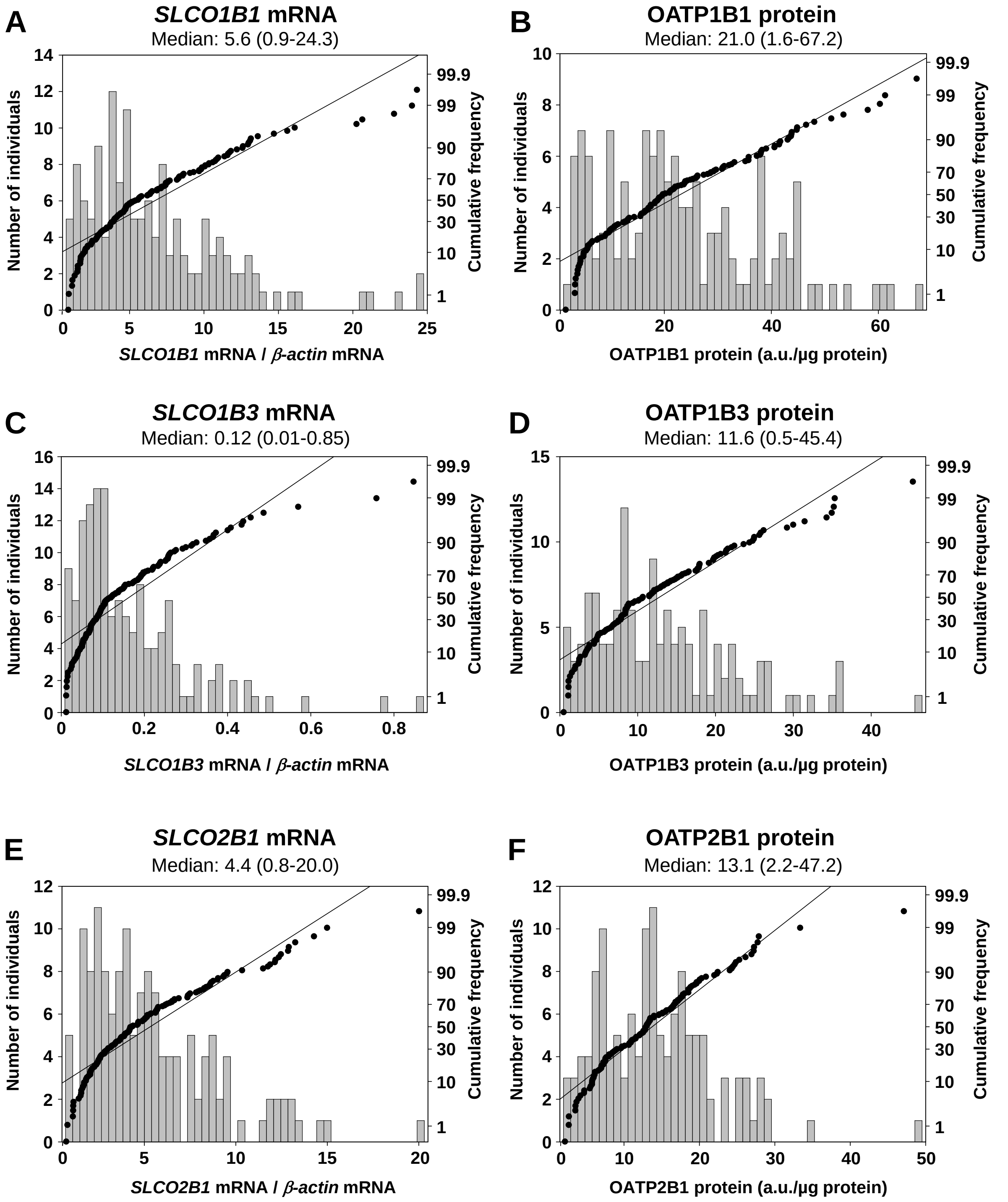

Figure S3. ***SLCO*** **genetic variants affect expression of hepatic OATPs and atorvastatin pharmacokinetics.** Box-whisker plots of (A) OATP1B1 protein expression in the non-cholestatic liver samples and (B) atorvastatin AUC in the healthy volunteers based on the number of alleles of *SLCO1B1*1b*, *SLCO1B1*14*, and *SLCO1B1*35*. OATP1B1 protein levels increase and atorvastatin AUC decreases per allele. Horizontal line: median; boxes: 25th-75th percentiles; whiskers: non-outlier range.

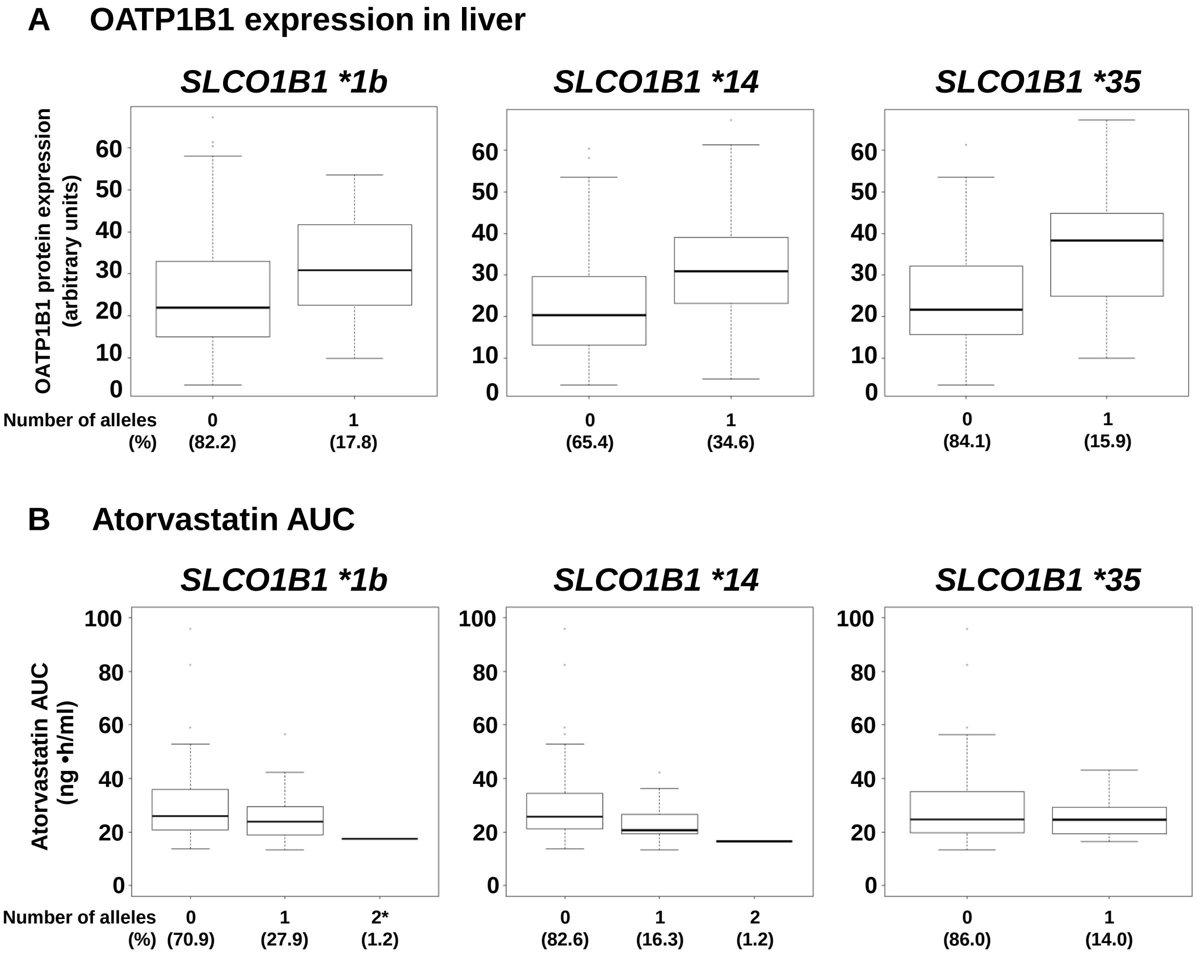

* "2" corresponds to the homozygous variant GG genotype (c.388A>G).

Figure S4. Immunolocalization of OATP2B1 and missense variants in transfected HEK cells and in human liver using a previously described antibody [29]. (A) All three variant OATP2B1 proteins were localized in the plasma membrane of transfected HEK cells. (B) Since exclusively homozygous variant liver samples are acceptable for analysis, data on localization could be only established for the variant OATP2B1-c.935AA showing sinusoidal hepatocyte membrane staining that was not different from localization of OATP2B1-reference sequence. Bars, 20 µm.

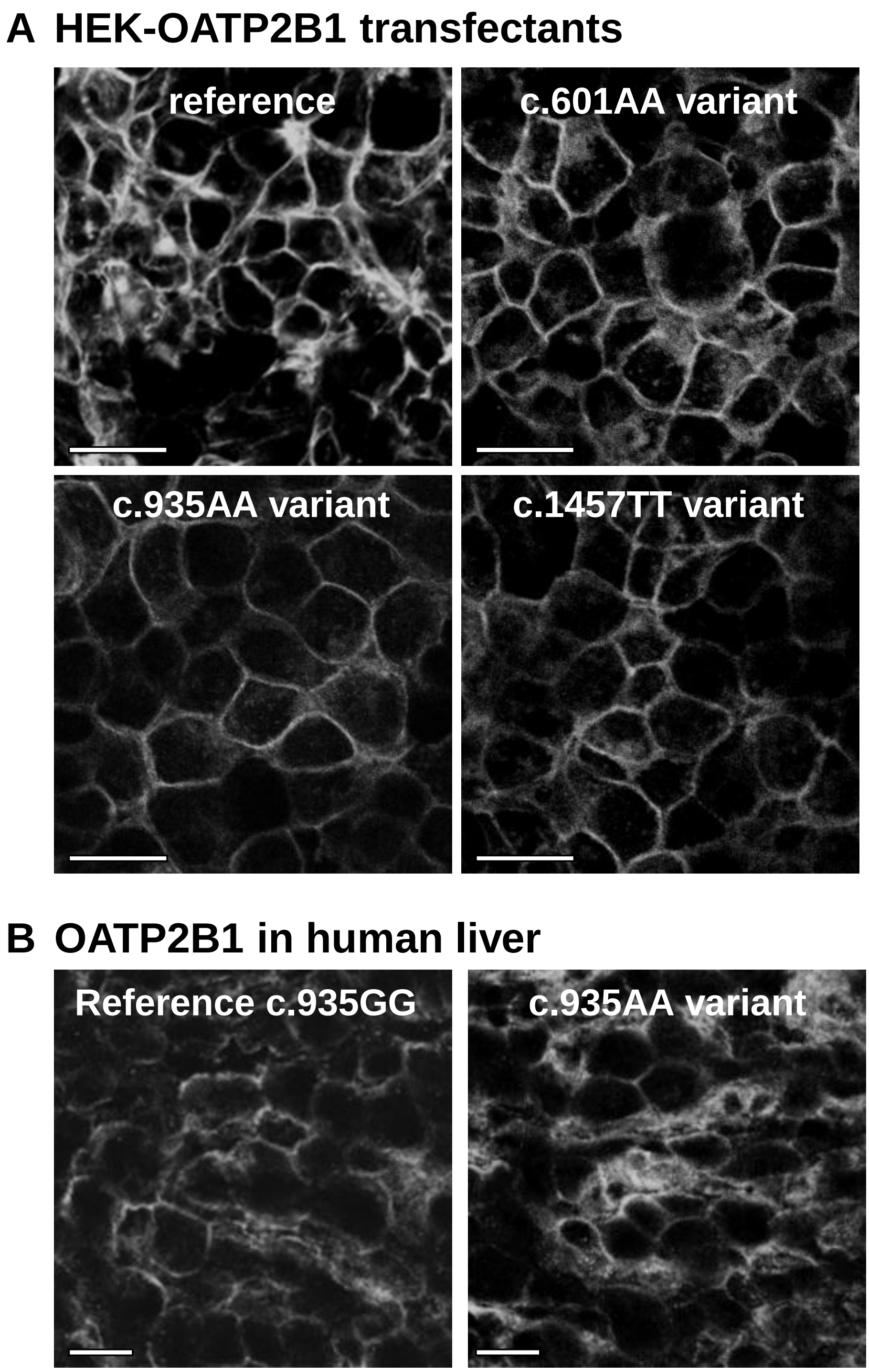

Figure S5. Functional characterization of OATP2B1 and 3 missense variants using atorvastatin, rosuvastatin, and the prototypical substrate estrone sulfate. Concentration-dependent uptake of atorvastatin (A), rosuvastatin (B), and estrone sulfate (C) by OATP2B1-reference (●), and 3 missense variants (c.601G>A ▼, c.935G>A ◆, c.1457C>T ■) using stably-transfected HEK cells. Data are means ± SE (n=3 experiments, each performed in triplicate).

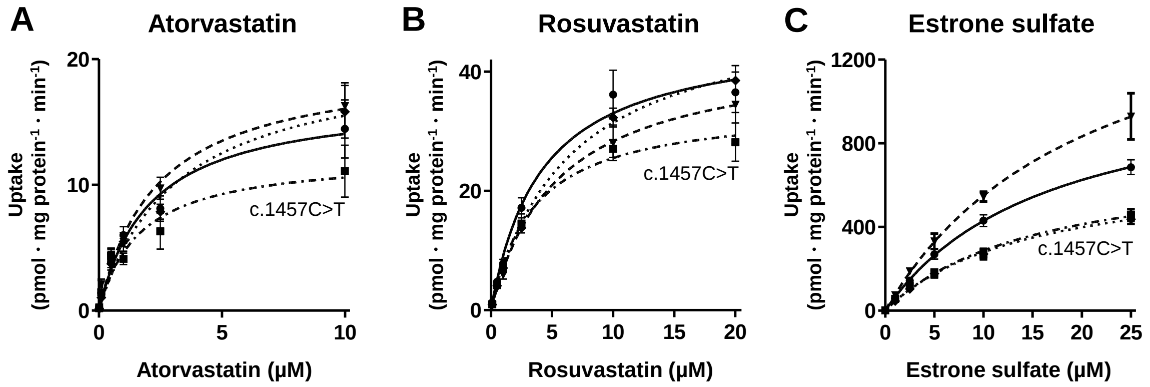

Figure S6. Immunolocalization of OATP1B1 in cryosections from genotyped human liver samples using a previously described antibody [30]. Compared with samples from carriers of the reference genotype c.388AA/c.521TT (A), OATP1B1 was correctly localized in the sinusoidal hepatocyte membrane of liver samples from carriers with the *SLCO1B1* haplotype c.388GG/c.521TT (B) and c.388GG/c.521CC (C). Bars, 20 µm.

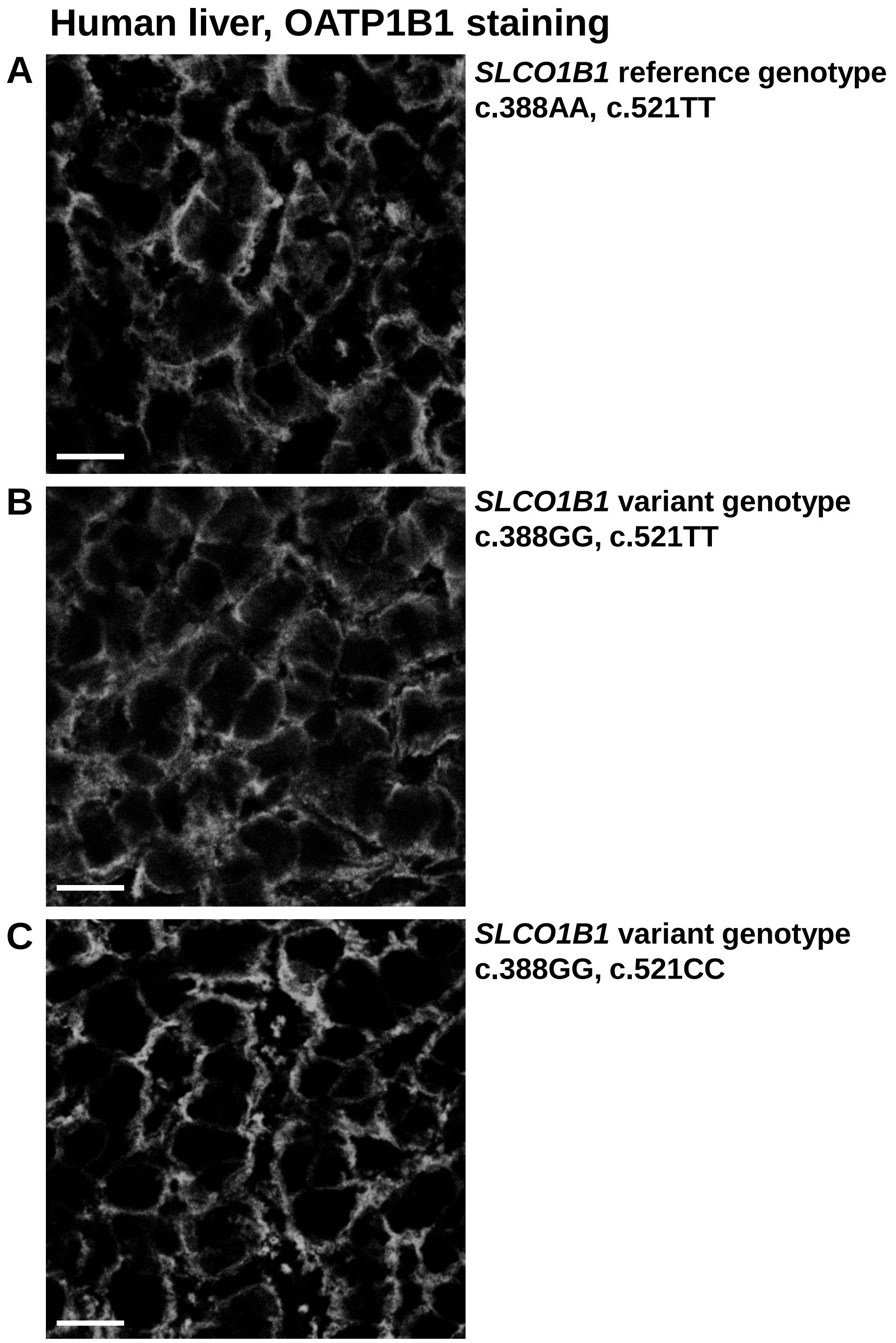

Figure S7. Prediction of secondary *SLCO1B1* mRNA structures using MFold. In each case, the optimal structure is shown and the initial ΔG value is given in kcal/mol. The variants c.388A>G and c.521T>C are marked in boldface.

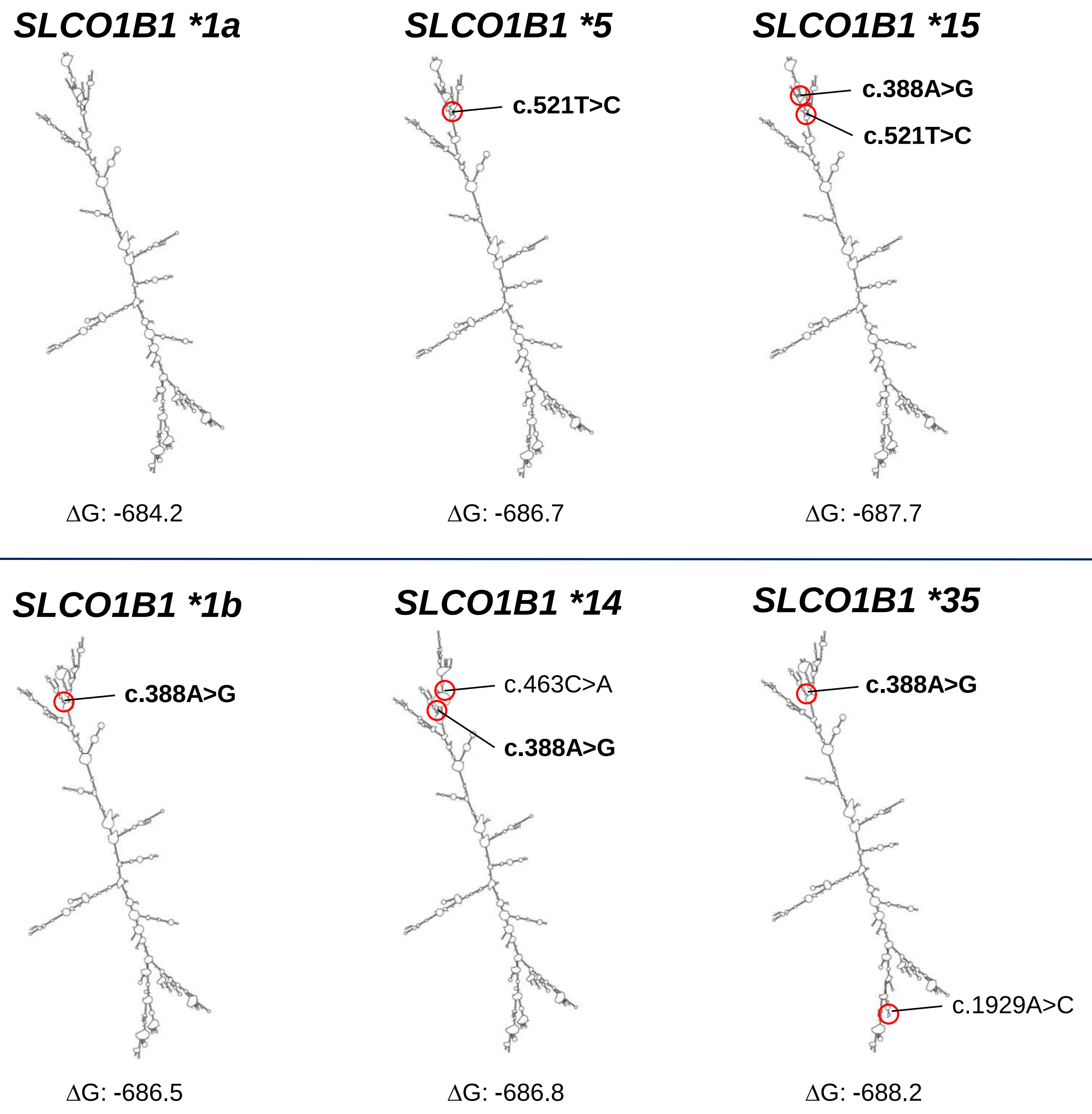

Figure S8. Transcriptional regulation of *SLCO* expression. (A) *In silico* analysis and identification of three high-scoring HNF1α binding sites in the promoter of *SLCO2B1* exon_1e, whose transcripts are highly abundant in human liver [47] compared to transcripts of the exon_1b promoter previously analyzed [18]. Boldface: bases fitting to the HNF1 consensus binding motif; numbers: position of motifs relative to the transcriptional start sites. (B) EMSAs with (+) or without (-) HNF1α protein showed specific complexes of HNF1α homodimers (arrow) with the oligonucleotides corresponding to the indicated HNF1 motifs.

A

**HNF1 consensus**	**GGTTAAT**	**N**	**ATTAACC**	
SLCO1B1 (-51/-38)	**GGTTAAT**	**C**	**AT**c**A**ctg	(+) strand
SLCO1B3 (-61/-47)	**GGTTAAT**	**C**	**AT**c**A**ttg	(+) strand
SLCO2B1 site 1 (-2204/-2190)	t**G**a**TAA**a	**T**	**ATT**c**A**ag	(-) strand
SLCO2B1 site 2 (-1576/-1562)	t**G**ca**AA**a	**A**	**ATTA**g**CC**	(-) strand
SLCO2B1 site 3 (-1522/-1508)	**GGTT**t**AT**	**T**	**ATT**tttt	(+) strand

B

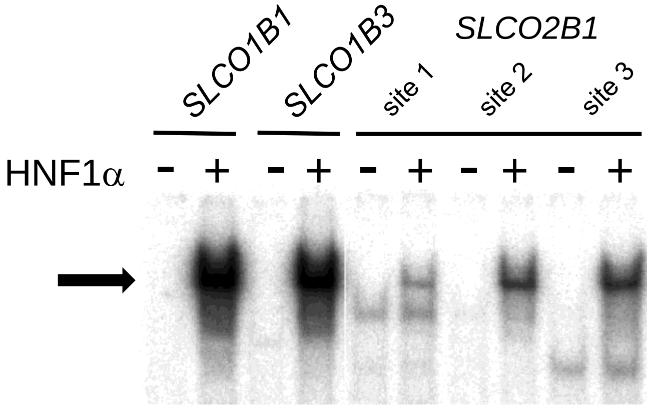

Supplement: Additional data file 1 — Additional methods, tables and figures [55-79]. [file gm405-S1.PDF]
